# Enhanced oligomerization of full-length RAGE by synergy of the interaction of its domains

**DOI:** 10.1038/s41598-019-56993-9

**Published:** 2019-12-30

**Authors:** Alexander Moysa, Dietmar Hammerschmid, Roman H. Szczepanowski, Frank Sobott, Michal Dadlez

**Affiliations:** 1 Institute of Biochemistry and Biophysics, PAN, Pawinskiego 5a, 02-109 Warsaw, Poland; 2 Biomolecular & Analytical Mass Spectrometry, University of Antwerp, Groenenborgerlaan 171, 2020 Antwerp, Belgium; 3 International Institute of Molecular and Cell Biology, Trojdena 4, 02-109 Warsaw, Poland; 4 Astbury Centre for Structural Molecular Biology and School of Molecular and Cellular Biology, University of Leeds, Woodhouse Lane, LS2 9JT Leeds, UK

**Keywords:** Structural biology, Membrane proteins

## Abstract

The pattern recognition receptor RAGE (receptor for advanced glycation end-products) transmits proinflammatory signals in several inflammation-related pathological states, including vascular diseases, cancer, neurodegeneration and diabetes. Its oligomerization is believed to be important in signal transduction, but RAGE oligomeric structures and stoichiometries remain unclear. Different oligomerization modes have been proposed in studies involving different truncated versions of the extracellular parts of RAGE. Here, we provide basic characterization of the oligomerization patterns of full-length RAGE (including the transmembrane (TM) and cytosolic regions) and compare the results with oligomerization modes of its four truncated fragments. For this purpose, we used native mass spectrometry, analytical ultracentrifugation, and size-exclusion chromatography coupled with multi-angle light scattering. Our results confirm known oligomerization tendencies of separate domains and highlight the enhanced oligomerization properties of full-length RAGE. Mutational analyses within the GxxxG motif of the TM region show sensitivity of oligomeric distributions to the TM sequence. Using hydrogen–deuterium exchange, we mapped regions involved in TM-dependent RAGE oligomerization. Our data provide experimental evidence for the major role of the C2 and TM domains in oligomerization, underscoring synergy among different oligomerization contact regions along the RAGE sequence. These results also explain the variability of obtained oligomerization modes in RAGE fragments.

## Introduction

Oligomerization is common among proteins, and two thirds of cellular proteins form oligomers^1^. Oligomer formation often seems crucial for membrane protein interaction with ligands and signal transduction^2–6^. Oligomerization is important for different types of receptors, such as immunoreceptors^7^ and T cell^8^, B cell^9^, and hormone receptors^10^. The receptor for advanced glycation end-products (RAGE) is a member of the immunoglobulin (Ig) superfamily of cell-surface receptors^11^. Apart from advanced glycation end-products, RAGE ligands include a variety of proteins, peptides, and nucleic acid chains^12–16^. RAGE signalling plays an important role in the adaptive immune response. As a crucial mediator of proinflammatory signalling, however, RAGE activation may also enhance a variety of pathophysiological states, such as arteriosclerosis, diabetes, and Alzheimer’s disease^17^.

The role of RAGE in a variety of pathologies makes it a potential target for therapeutic intervention. However, like other membrane proteins, RAGE presents a significant challenge in structural studies. Its inherent oligomerization tendencies, the dynamic nature of its domains and the need for detergents or lipids to maintain protein solubility render RAGE intractable to many biophysical techniques. In contrast, native mass spectrometry (Native MS) and hydrogen-deuterium exchange monitored by MS (HDX-MS) allow acquisition of structural information for oligomerizing proteins and protein–protein/ligand interactions in the presence of membrane-mimicking detergents or lipids^18^.

**Figure 1:**
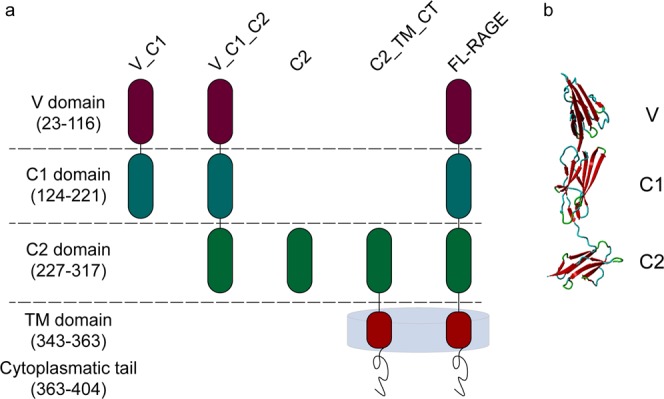
(**a**) Domain composition of full-length RAGE and different truncated variants used in this work. RAGE consists of the N-terminal V domain followed by two C-domains (C1, C2) of immunoglobulin-like fold, a transmembrane region (TM), and a cytoplasmic tail (CT). (**b**) X-ray structure of V_C1_C2 4YBH^16^

RAGE contains three extracellular domains – a V-type Ig domain (residues 23–116) and two C-type Ig domains (C1 and C2) (residues 124–221 and residues 227–317, respectively), a transmembrane (TM) helix (residues 343–363), and a short cytoplasmic tail (residues 363–404) (Fig. 1a). Each extracellular domain of RAGE contains two cysteines forming intramolecular disulphide bridges within a domain^19–21^. Cysteines of the C2 region may also form intermolecular bridges, stabilizing a RAGE dimer^14,19^. Structural studies of the extracellular part of RAGE (V_C1_C2) have shown that the V and C1 domains form an elongated structural unit connected with the C2 domain by a flexible linker (Fig. 1b)^16,22^.

Intrinsic oligomerization tendencies have hampered the classic approaches to elucidating the structure of this membrane-embedded protein complex in its full-length version. Instead, truncated variants, covering different regions of the extracellular part of RAGE and missing the TM region have been used. For instance, X-ray structures of isolated domains V_C1^13^ and V_C1_C2^22^ have been used as a basis for molecular modelling of the complex. Covalently stabilized oligomeric forms of subdomains also have been employed, cross-linked either by an engineered di-tyrosine bond^23^ or by standard chemical cross-linking in the V_C1_C2 construct, followed by molecular modelling^20^. In result of this work different modes of oligomerization were proposed, without consensus. Because electrostatic charges are unevenly distributed between the domains, pI values in different constructs are widely disparate (V_C1, pI = 9.9; V_C1_C2, pI = 7.8; C2, pI = 4.2). These differences may lead to a variety of arrangements when domains are studied in separation. Therefore, attempts to verify the obtained models using full-length RAGE that includes all potential interacting regions are crucial for revealing the native pairing and stoichiometry of the RAGE oligomers.

## Results

### RAGE constructs

To examine the role of different human RAGE domains in oligomerization, we compared the oligomerization patterns in full-length RAGE (FL_RAGE), covering the three extracellular domains, the TM region, and the intracellular tail with its four, differently truncated variants (V_C1, V_C1_C2, C2, and C2_TM_CT) (Fig. 1a). The two C-terminally truncated variants, V_C1 and V_C1_C2, contained the extracellular domains of RAGE. C2_TM_CT represented the N-terminally truncated variant, containing the C2, TM, and cytoplasmic tail, while C2 contained only the extracellular domain C2. Proteins were obtained by overexpression in *E. coli* and subjected to *in vitro* studies of their oligomerization status and structural dynamics by native MS, analytical ultracentrifugation (AUC), size-exclusion chromatography (SEC) coupled with multi-angle light scattering (MALS) and HDX-MS.

### Disulphide bonds in FL_RAGE

**Figure 2:**
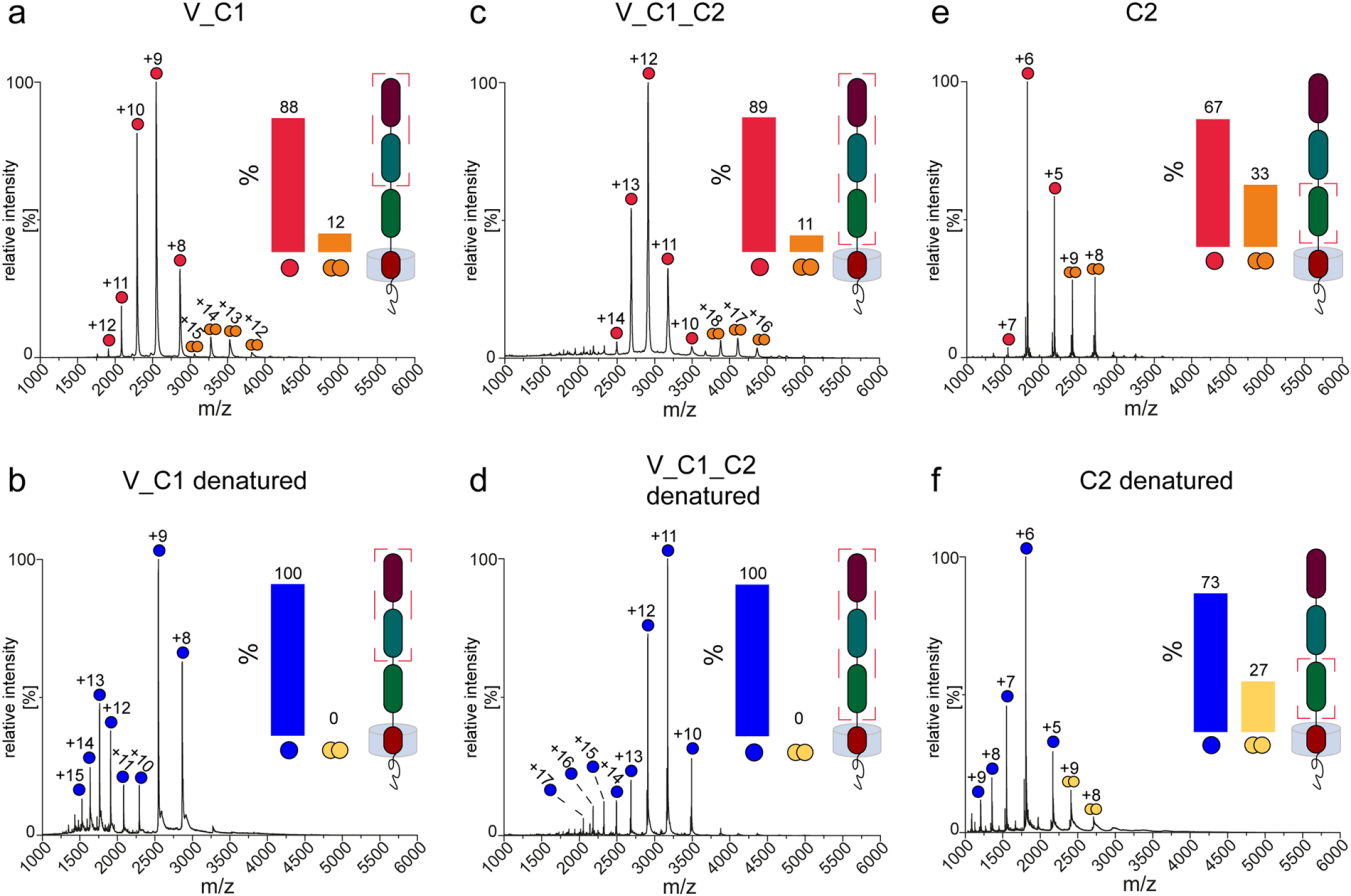
Mass spectra of V_C1 (**a**,**b**), V_C1_C2 (**c**,**d**), and C2 (**e**,**f**) introduced into the mass spectrometer under non-denaturing conditions (**a**,**c**,**e**) and denaturing conditions (**b**,**d**,**f**). Monomeric and dimeric species including their corresponding charge state are assigned accordingly for the different protein variants. The bar diagrams in the inset show the relative amount of monomers and dimers, respectively. The illustration highlights the composition of each construct in relation to the full-length protein by a red frame.

Disulphide linkages significantly influence protein structure and function. Incorrectly paired disulphide bonds trigger alteration in the three-dimensional structure followed by a resultant change in protein properties. Therefore, obtaining native disulphide bonds is a crucial moment in protein production (see “Protein production” section in Supplementary information). It has been established before in numerous studies^13,14,19–21^ that cysteines within V and C1 domains form only intradomain disulphide bonds while cysteines within the C2 domain can form intra- and interprotein disulphide bridges. Both variants of linkages between cysteines of C2 domains were observed in our study for C2 and C2_TM_CT samples (Figs. 2f and S4). In contrast, for the V_C1 protein, where the C2 domain was omitted, our results revealed only non-covalent dimers (Fig. 2a,b) which is in agreement with the previous results^13,21,24^. Apparently, the tendency of cysteine linkages between C2 domains caused the formation of high-order disulphide mediated oligomers of the full-length RAGE and V_C1_C2 during *E. coli* cytoplasmic expression (Fig. S2c). In order to overcome this problem, we used a periplasmic variant of *E. coli* expression, where intraprotein disulphide bonds were formed predominantly. For confirmation of the correct FL_RAGE disulphide bond formation, we used a MS-based strategy (Supplementary Fig. S1). Based on previous structural studies of extracellular domains, three intradomain disulphide bonds (linking residues 38–99, 144–208, and 259–301) were expected^14,20,21^. Our results (Supplementary Table S1) confirmed the correct disulphide bond pairing in FL_RAGE. For V_C1_C2 protein, we used the same production protocol as for the FL_RAGE. To further confirm the structural identity of the V_C1_C2 used here and obtained before, we have verified that the hydrogen-deuterium exchange pattern for V_C1_C2 protein was the same (Fig. S6a) as demonstrated in a previous study^23^. Therefore, we conclude a proper formation of the expected structure of V_C1_C2 protein necessitating the correct formation of disulphide bridges.

## Oligomerization States of FL_RAGE

### SEC-MALS

**Figure 3:**
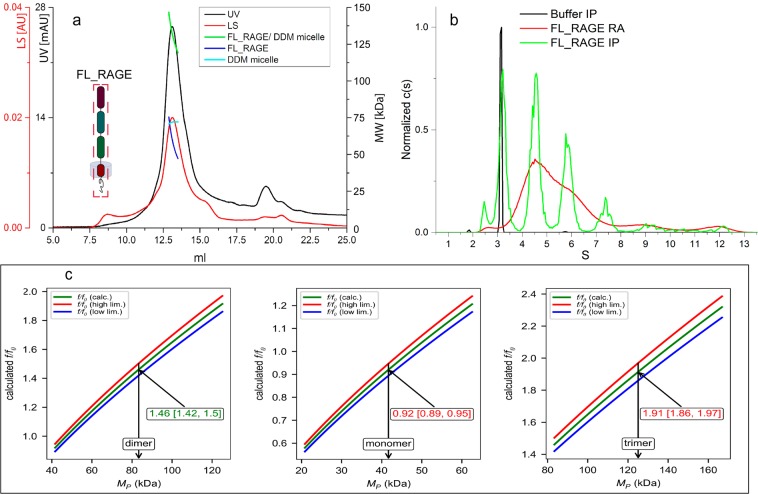
SEC-MALS and AUC analysis of full-length RAGE. (**a**) SEC-MALS of FL_RAGE. Chromatograms show the detector readings of the LS and UV detectors in red and black, respectively. Scales for LS and UV detectors are shown on the left-hand axis; scale on right-hand axis represents the molecular mass. The green, blue, and cyan lines across the peaks indicate calculated molecular masses of the protein/detergent complex (green), the protein (blue), and the attached detergent micelle (cyan). (**b**) Sedimentation velocity experiment of DDM-solubilized FL_RAGE (absorption at 280 nm in red and interference in green). The black trace represents the interference data of the buffer including DDM micelles. (**c**) Example of the analysis of the dataset of FL_RAGE (panel b) using type f/fo GUSSI Membrane Protein Calculation module. Red, green, and blue lines represent the high, optimal, and low limits of f/fo for the protein/micelle fraction, respectively (see text). The pictures show the analysis of the 5.8S peak and indicate the presence of a dimer. The 4.4S and 7.4S peaks represent monomers and trimers, respectively (see Fig. S5 in Supplementary information).

In order to maintain FL_RAGE in solution, we applied a detergent environment capable of mimicking the native lipid environment in which the TM region is normally embedded. Therefore, FL_RAGE was purified and characterized in the presence of the non-ionic detergent n-Dodecyl β-D-maltoside (DDM). The SEC-MALS analysis of FL_RAGE in the presence of DDM revealed a well-defined peak (Fig. 3a). In the case of the protein–detergent complex, however, the interpretation of the corresponding masses is not straightforward. The amount of detergent bound to the protein may differ depending on both the protein structure and the nature of the detergent. Therefore, the mass ratio between the protein and detergent in the micelle can vary dramatically. A dedicated approach is required in the data analysis step, which considers the presence of the detergent^25^. The “protein conjugate” module of ASTRA software (Wyatt Technologies) enables the calculation of the molecular weight of the whole complex as well as its protein component and the detergent micelle mass associated with the protein.

The SEC-MALS chromatogram of FL_RAGE (Fig. 3a) showed a main protein peak eluting at ~13 ml. The molar mass of the complex, estimated by ASTRA, was heterogeneous across the peak, spanning the range of masses of the protein–detergent micelle complex (~120–140 kDa). The calculated molar mass of the protein was also heterogeneous (~47–75 kDa), indicating a substantial non-monomeric fraction in the samples of FL_RAGE (the expected molecular mass of FL_RAGE is 41.8 kDa).

### AUC

The sedimentation velocity of FL_RAGE (Fig. 3b) was investigated by monitoring the absorbance and interference data. The absorbance at 280 nm displayed a broad signal between 3.5S and 8.0S, with the maximum around 4.4S and a trace amount of higher molecular weight species. At the same time, the interference, characterized by a better spatial resolution as compared to the absorption, showed three well-defined peaks (4.4S, 5.8S, and 7.4S) located in the same range of 3.5–8.0S. The additional peak at 3.2S clearly corresponded to the interference boundary observed for the buffer. This peak was not detected when the buffer alone was investigated with absorbance (data not shown) indicating the presence of free DDM. Similar signal was also observed by other authors^26^. Interpreting these findings in terms of the oligomerization status of FL_RAGE required a more detailed analysis. For this purpose, we used the f/f_o_ analysis procedure described in the GUSSI Membrane Protein Module package by Brautigam^27^. Following this, the results indicated that the 4.4S, 5.8S, and 7.4S peaks corresponded to monomer, dimer, and trimer, respectively, as only in this case the f/f_o_ values were within the allowed range of 1.2–1.7. The assumption of another degree of oligomerization led to f/f_o_ values deviated from the permissible range: >1.9 and <1 for higher order and smaller oligomers, respectively, which is not possible. Figure 2c illustrates this type of analysis based on peak number 2 (5.8S). These results of the AUC experiment revealed the presence of a mixture of monomers, dimers, trimers and even a small fraction of higher order oligomers, which is in good agreement with the SEC-MALS result. Obviously, a rapid exchange between monomeric and dimeric species resulted in a broad peak at 280 nm.

### Native MS

**Figure 4:**
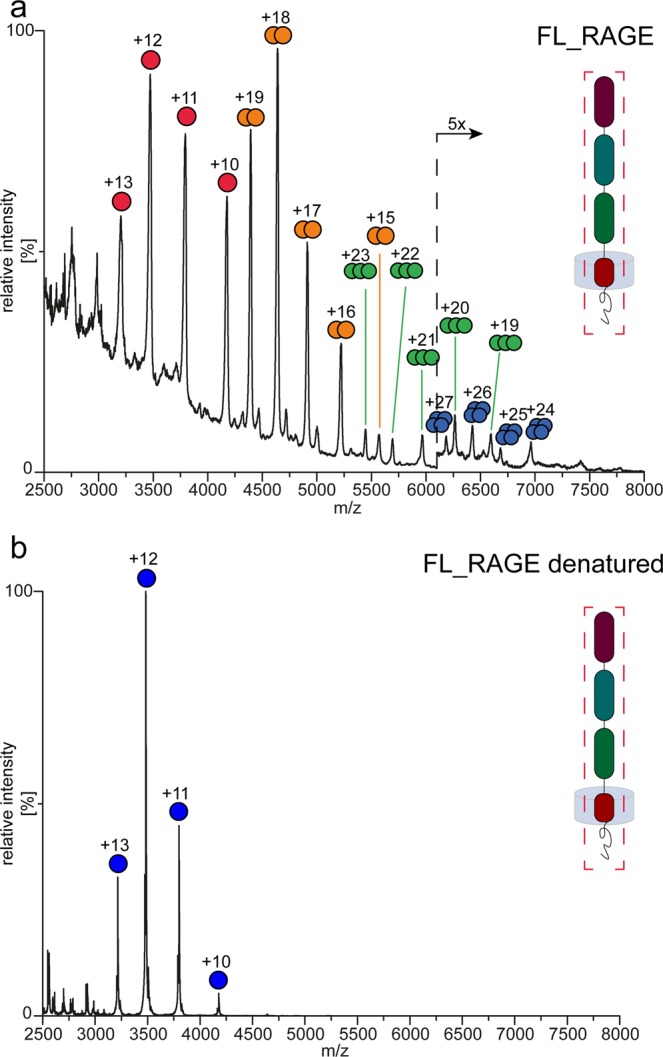
Mass spectra of FL_RAGE under native (**a**) and (**b**) denaturing conditions. Peaks are marked by colored dots and the corresponding charge state for monomers and multimers of FL_RAGE accordingly.

For native MS analysis, FL_RAGE was solubilized in DDM or in Triton X-100 at a concentration of two times the critical micelle concentration (CMC). Despite the stability of FL_RAGE in the presence of DDM during protein purification, the buffer exchange to an MS-friendly ammonium acetate (AmAc) solution, which also contained 2x CMC of DDM, led to protein precipitation, even at a quite high concentration of AmAc (1M). The precipitation of FL_RAGE was not observed when Triton X-100 (2x CMC) was used instead of DDM. Following this, the native MS spectrum acquired at 600 mM AmAc revealed monomeric, dimeric, trimeric, and even tetrameric signals (Fig. 4a). Both monomeric and dimeric species are close to equilibrium and accounted for the vast majority of the signal. Interestingly, the purification of FL_RAGE using SEC instead of ion exchange chromatography enabled a better preservation of higher-order oligomers, e.g. trimers and tetramers (Fig. S3), with however some impurities (unidentified contaminants) and a more challenging membrane protein liberation in the gas phase (poor resolution). The addition of 0.1% formic acid and 50% acetonitrile to FL_RAGE led to the disappearance of signals stemming from oligomeric species (Fig. 4b), indicating a non-covalent character of the oligomerization. As native MS of membrane proteins depends on a collision-activated liberation of the protein from the detergent micelle cluster, we required a minimum of (15V) 30V collision energy along with an activation of 200V in the sampling cone in order to observe (monomeric) oligomeric species of FL-RAGE in the spectrum.

## Oligomerization of truncated variants of RAGE: V_C1, V_C1_C2, and C2

### SEC-MALS

**Figure 5:**
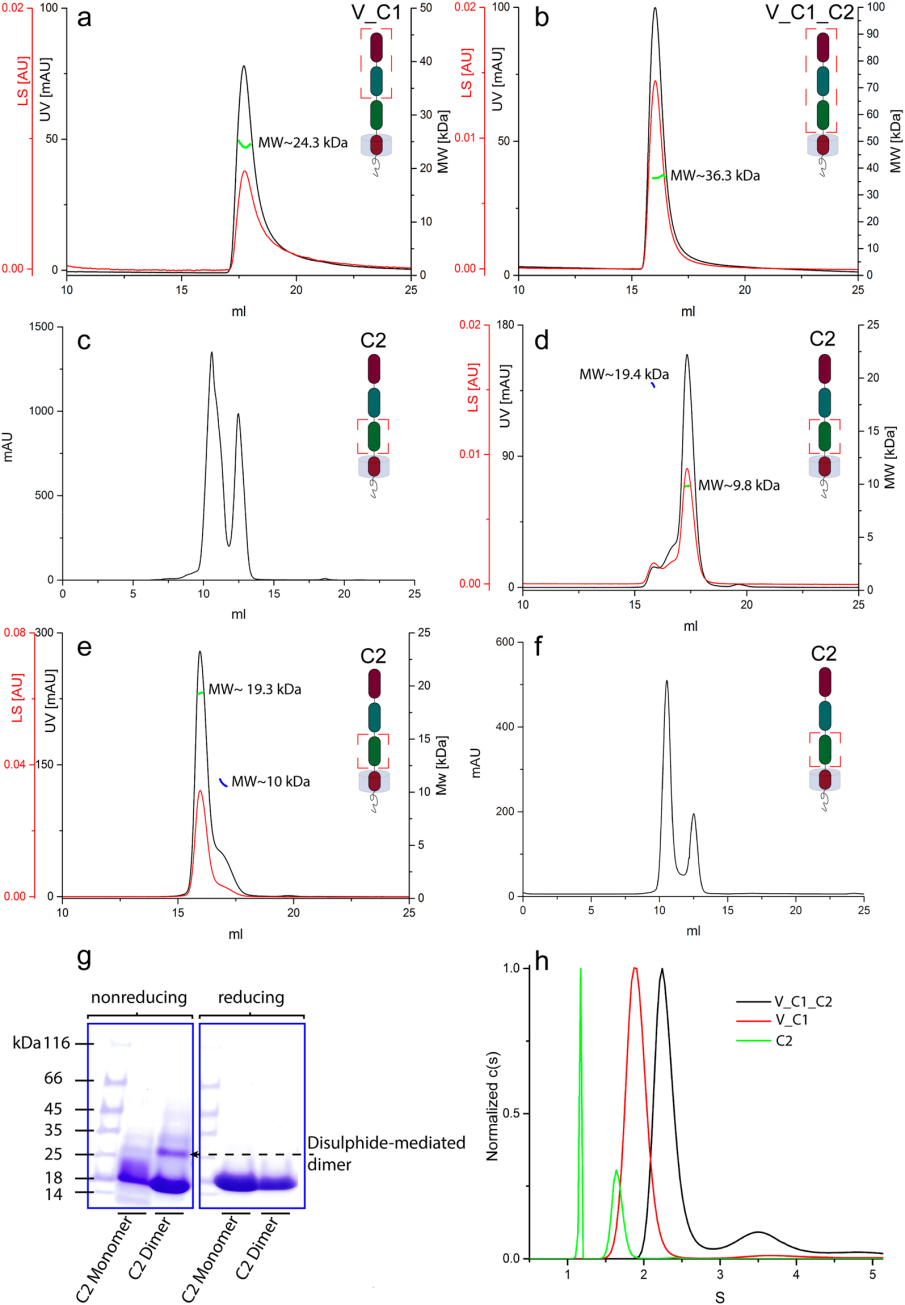
SEC, SEC-MALS, and AUC analysis of the constructs V_C1, V_C1_C2, and C2. (**a**–**f**) SEC/SEC-MALS chromatograms show the detector readings of the LS and UV in red and black, respectively. Scales for LS and UV detectors are shown in the left-hand axis; and the calculated molecular mass on the right-hand axis. (**a**) SEC-MALS of V_C1. (**b**) SEC-MALS of V_C1_C2. (**c**) SEC of C2. (**d**) SEC-MALS of the monomeric fraction of C2. (**e**) SEC-MALS of the dimeric fraction of C2. (**f**) SEC of C2 upon the addition of a reducing agent (1 mM TCEP). (**g**) SDS-PAGE of the monomeric and dimeric fractions of C2 under reducing and non-reducing conditions (full-length gels see Fig. S2 in Supplementary information). (**h**) AUC (sedimentation velocity profiles) of V_C1, V_C1_C2, and C2.

The elution profiles of V_C1 and V_C1_C2 in SEC-MALS showed homogeneous peaks (Fig. 5a,b). The calculated molecular masses for V_C1 and V_C1_C2 revealed values of 24.3 and 36.3 kDa, respectively. These values are in close proximity to the molecular weight of monomeric V_C1 (MW_theoret_ = 22.8 kDa) and V_C1_C2 (MW_theoret_ = 34.9 kDa). The elution profile of C2 displayed two peaks during SEC purification (Fig. 5c). Both fractions were collected and re-chromatographed separately for the determination of the molecular mass by MALS. The calculated M̄w of ~10 and ~19 kDa corresponded to the monomer (MW_theoret_ = 10.7 kDa) and dimer (MW_theoret_ = 21.4 kDa) of C2, respectively (Fig. 5d,e). A rechromatography of the dimer fraction showed a limited monomeric content (Fig. 5e), indicating that the dimer of C2 was stable.

As the C2 variant contains two cysteines, we considered the possibility of a covalently formed dimer. In order to verify the type of dimerization, we performed SEC in the presence of a reducing agent (Fig. 5f), which still showed a reasonable fraction of dimers. The SDS-PAGE analysis of the dimeric fraction (Fig. 5g) under non-reducing conditions showed a minor dimeric fraction, confirming the disulphide-mediated, non-covalent character of the dimer. These results underscore the strong intrinsic oligomerization properties of the C2 domain.

### AUC

The sedimentation velocity of V_C1, V_C1_C2, and C2 was investigated by monitoring the absorbance at 280 nm. In the case of V_C1_C2, the c(s) distribution showed two peaks: a main peak (72% of the signal) at 2.3S and a minor peak (17% of the signal) at 3.58S corresponding to the monomer and dimer, respectively (Fig. 5h). The experimental-derived molecular masses of 32.4 kDa and 62.7 kDa were in good agreement with the theoretical sequence-based masses, namely 34.9 kDa for the monomer and 69.8 kDa for the dimer. Trace amounts of even higher molecular weights were observed with however less than 1.5% of the total signal area. This result is in excellent agreement with previous AUC results^28^. The contradicting SEC-MALS result – only a monomeric fraction was present – may be the result of a dilution effect during SEC. V_C1 only displayed a monomeric peak at 1.91S (93% of the total signal) (Fig. 5h), with excellent agreement of the experimental and theoretical masses: 22.5 kDa versus 22.9 kDa, respectively.

The standard c(s) distribution of C2 revealed two main peaks (Fig. 5h). In contrast to previous experiments, however, the resulting masses did not agree with the theoretical values calculated from the amino acid sequence. In order to solve this problem, a Sedfit “Continuous c(s) with bimodal f/f_0_” procedure was used. During the calculations, we considered a change in the coefficient f/f_0_ from 1.39 in the case of the monomer to 1.65 in the case of the dimer, which enabled a good match between experimental and theoretical masses: 9.7 kDa for the monomeric peak (MW_theoret_ = 10.7 kDa) and 21.1 kDa for the dimeric peak (MW_theoret_ = 21.4 kDa). This finding suggests that dimerization resulted in the elongation of protein shape. The AUC of C2 showed the prevalence of a dimer (over 65% of the total signal), which is in accordance with the SEC result (Fig. 5c).

### Native MS

We also conducted native MS experiments for V_C1, V_C1_C2, and C2. The spectrum of V_C1 (Fig. 2a) revealed the prevalence of monomeric species accompanied by prominent dimeric signals (~12%). V_C1_C2 showed approximately the same fraction of dimers (~11%) (Fig. 2c), with well-resolved charge state series corresponding to both monomeric (predominant) and dimeric states (Fig. 2c). The spectrum of C2 (Fig. 2e) was also dominated by monomeric signals, but the dimeric signals was much higher in intensity (~33%) compared to V_C1 and V_C1_C2. This pattern suggested a relatively higher tendency for the formation of C2 dimers, which is in accordance with the results obtained from AUC and SEC experiments (Fig. 5).

As all variants contain cysteine residues, we also conducted MS experiments under denaturing conditions to verify the non-covalent character of the oligomerization. Upon the addition of denaturants (50% acetonitrile and 0.1% formic acid), which causes the disruption of non-covalent interactions, the dimeric signals in V_C1 (Fig. 2b) and V_C1_C2 (Fig. 2d) disappeared entirely. Moreover, the intensity of peaks corresponding to highly charged monomers increased, indicating the denaturation of the proteins and highlighting the non-covalent character of the dimerization in these constructs. The spectrum of C2 (Fig. 2f) under denaturing conditions however still exhibited a significant fraction (~27%) of dimeric peaks. A covalent dimerization in the C2 domain, as observed in Fig. 5g, is possible, although the MS data do not allow a clear statement in this respect. Overall, native MS experiments confirmed increasing oligomerization tendencies for the C2 domain in comparison with V_C1 or V_C1_C2 as well as for FL_RAGE in comparison with V_C1 or V_C1_C2. Hence, the C2 and TM domains seems to play an important role in terms of the oligomerization of RAGE. Native oligomerization should thus be assessed in the context of the full-length protein, as truncated constructs tend to distort native oligomeric patterns.

## Oligomerization of the N-truncated variant of FL_RAGE (C2_TM_CT)

### SEC-MALS

**Figure 6:**
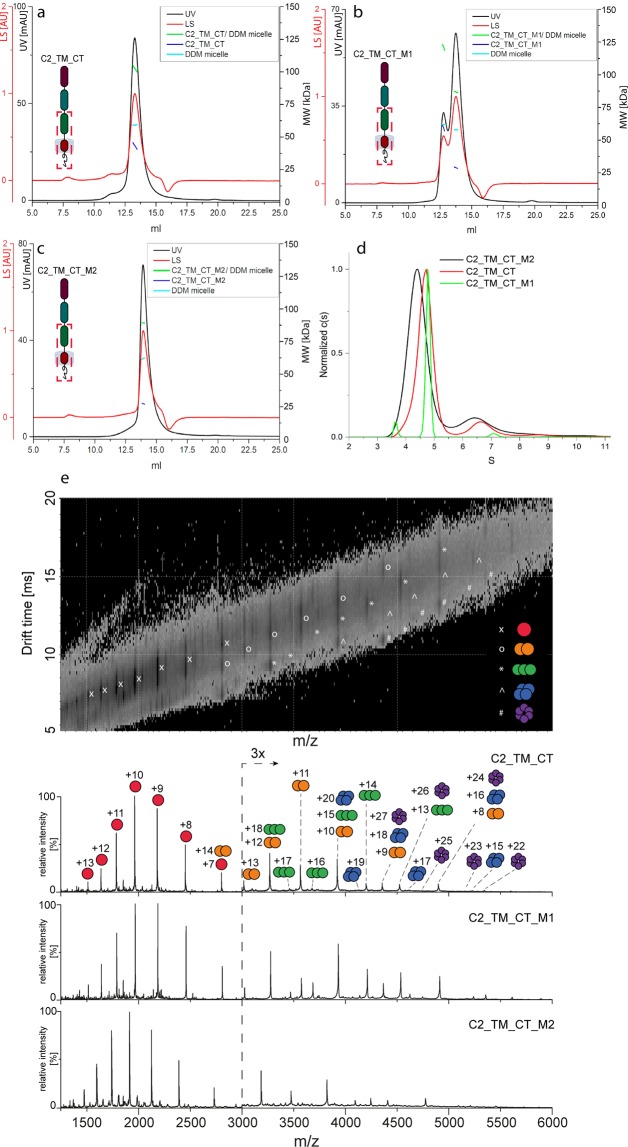
SEC-MALS, AUC and native MS analysis of C2_TM_CT wild-type and mutants. (**a**) SEC-MALS of C2_TM_CT. (**b**) SEC-MALS of C2_TM_CT_M1. (**c**) SEC-MALS of C2_TM_CT_M2. Chromatograms show the detector readings of the LS and UV in red and black, respectively. Scales for LS and UV detectors are shown in the left-hand axis; and calculated molecular mass on the right-hand axis. (**d**) AUC (sedimentation velocity profiles) of C2_TM_CT and mutants. (**e**) Native mass spectra of C2_TM_CT wild-type and the mutant variants (1 & 2) solubilized in DDM micelles with the 2D drift plot of the wild-type in the top panel. In the case of overlapping oligomeric species, the drift plot can help to identify the correct oligomeric states that are represented by the same m/z peak. Monomeric and oligomeric species including their charge states are assigned accordingly. Corresponding peaks of C2_TM_CT_M2 are slightly shifted to lower m/z values due to the deletion of six amino acids.

For the entire C-terminal part of RAGE (C2_TM_CT), SEC-MALS was performed in the presence of DDM and the data were analyzed according to the “protein conjugate” method (Fig. 6a). The chromatogram displayed a protein peak at ~13.3 ml. The ASTRA analysis revealed an average molecular mass of the protein of 43 kDa, indicating to the formation of a dimer (monomeric mass: 19.6 kDa).

### Native MS

Native MS analysis of C2_TM_CT was performed in the presence of DDM at 2x CMC and 200 mM AmAc (Fig. 6e). The spectrum highlighted monomers, dimers, trimers, and even small amounts of tetramers and hexamers. Since mass spectrometry measures the m/z ratio of the analyte, different oligomers can result in the same m/z values which may cause partial overlap in the spectrum. In the case of overlapping oligomers, ion mobility measurements can help to identify the underlying species (Fig. 6e), though the analysis of peak intensities remains difficult. Nevertheless, the fact that higher-order oligomers (i.e. trimers, tetramers, and hexamers) were only observed for C2_TM_CT but not for V_C1, V_C1_C2, and C2 - all species were acquired at the same concentration – suggests an increased oligomerization tendency in the TM domain of the protein. However, based on intensities of the oligomeric signals in native MS of FL-RAGE and C2_TM_CT, it is difficult to compare these results directly, as the collision-activated liberation depends on the size of the complex as well as the detergent used for solubilization, and both varied in these constructs. Therefore, the higher intensity of FL_RAGE oligomeric signals may demonstrate an enhanced oligomerization of FL_RAGE. On the other hand, lower intensity of the oligomeric signals in C2_TM_CT may originate from the dissociation of a substantial fraction of oligomers, indicated by a relatively broader charge state distribution for monomers of C2_TM_CT (Fig. 6e) compared to FL_RAGE (Fig. 4a). The spectrum of C2_TM_CT (Fig. S4) under denaturing conditions exhibited not only dimeric but also tetrameric and hexameric species. These higher-order oligomers (tetramer and hexamer) might originate from strong non-covalent interactions - membrane proteins are generally known as more challenging to denature^29^ – or from interprotein disulphide bonds.

### Mutational analysis of the TM domain

As previous experiments indicated a direct involvement of the TM region in the oligomerization of RAGE, we attempted to confirm this hypothesis by performing mutational analyses. The presence of a GxxxG motif (two glycines separated by any three amino acids) in the TM region (LGTLALALGILGGLGTAALLIGVILW) is an indicator of possible TM helix–helix interactions^30,31^. The GxxxG motif mediates the packing of TM alpha helices by permitting close association and thus allowing interactions between the helix backbones as well as the side chains of surrounding residues^32^. Therefore, we directed our attention to this region and designed two variants of C2_TM_CT with mutations affecting the GxxxG motif. In the first variant, four glycines in the region of the GxxxG motif were mutated to alanine (Mutant 1). In the second variant, six amino acids that cover the GxxxG motif were deleted (Mutant 2) (Supplementary Table S2).

The SEC-MALS experiments indicated the influence of mutations on the oligomerization pattern of both mutants. The chromatograms showed an increased retention volume (13.8 ml) of the major peaks of C2_TM_CT_M1 and C2_TM_CT_M2 as compared to C2_TM_CT (13.3 ml), indicating lower molecular masses of the corresponding peaks (Fig. 6a–c). The calculated average molecular masses of the protein, detergent micelle, and protein–detergent micelle complex were at 27, 62, and 89 kDa, respectively, and in close proximity between both mutants. The obtained molecular mass of the protein fits neither to the monomer (MW_theoret_ = 19.6 kDa, Mutant 1; 19.1 kDa, Mutant 2) nor to the dimer. Therefore, it is plausible that this peak contained a mixture between a monomer and dimer in fast equilibrium, resulting in an experimental molecular mass of 27 kDa. In addition to the main peak (13.8 ml), the glycine-to-alanine substitution in Mutant 1 resulted in an additional peak at 12.7 ml, with a calculated molecular mass of 59 kDa, which corresponds to the trimer (MW_prot/det_ = 121 kDa; MW_det_ = 61 kDa).

AUC (Fig. 6d) also showed differences in the sedimentation of C2_TM_CT and its variants. The highest value of c(s) = 4.8S was observed for the main peak of C2_TM_CT_M1. This peak contained 87.4% of the total signal (280 nm) and was surrounded by two other minor peaks, each with less than 7% of the signal. The C2_TM_CT was represented by a slightly slower boundary at 4.7S (85.0% of the signal) and the second one at 6.7S (11.9% of signal). The c(s) distribution of C2_TM_CT_M2 revealed a main peak with the lowest c(s) at 4.4S (80.1% of the total signal) and a second, much smaller peak at 6.8S (18.1% of the signal). The lower c(s) of C2_TM_CT_M2 indicated, on average, a lower oligomerization compared to C2_TM_CT and C2_TM_CT_M1. Apparently, the G/A substitution in C2_TM_CT_M1 resulted in a slightly enhanced oligomerization, as the main peak was also narrower than those of C2_TM_CT and C2_TM_CT_M2.

The two C2_TM_CT mutants were also subjected to native MS experiments In comparison with wild-type C2_TM_CT, the native MS measurements displayed an increased intensity of oligomeric signals for C2_TM_CT_M1, while wild-type and C2_TM_CT_M2 did not differ significantly (Fig. 6e). Overall, AUC, SEC, and native MS analysis of the two mutants indicated that the G/A substitutions might enhance the oligomerization, while the deletion of the GxxxG motif decreases it. Oligomeric distributions proved to be sensitive to substitutions in the TM region, confirming its role in oligomerization.

### Amide H/D exchange and differences in dynamics between V_C1_C2 and FL_RAGE

In order to investigate structural differences between V_C1_C2 and FL_RAGE, we measured the H/D uptake in both proteins. As of the enhanced oligomerization in FL_RAGE, we expected changes in structural dynamics upon the presence of the TM domain, revealing protein regions involved in the TM-mediated oligomerization. Therefore, we compared the exchange in a set of peptic peptides derived from V_C1_C2 and FL_RAGE. Overall, 71 peptides with satisfactory signal-to-noise ratio and a 78% coverage of the V_C1_C2 sequence were reproducibly identified after brief pepsin digestion (Supplementary Table S3). The intertwined pattern of protected and exposed structural elements of V_C1_C2 – as observed somewhere else^23,33^ and also in this work (Fig. S6a, blue symbols) - was fully retained in FL_RAGE (Fig. S6a, red symbols). The observed differences between V_C1_C2 and FL_RAGE are visualized in the differential plot, covering the extracellular part of the protein (Fig. S6b). Of interest, only local and no global changes in the protection levels were observed.

**Figure 7:**
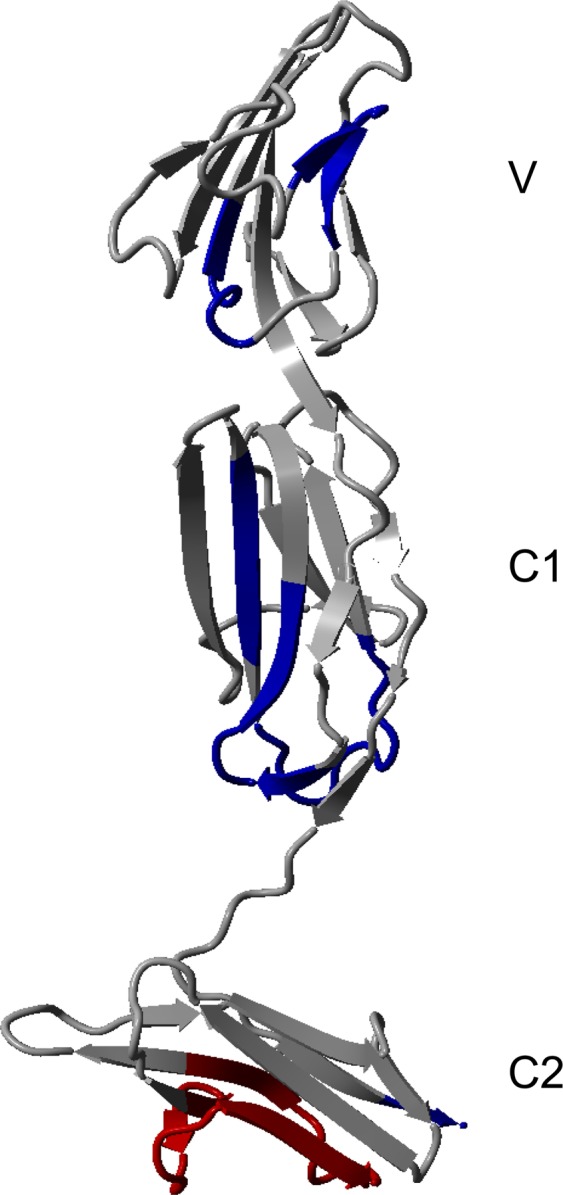
H/D exchange differences between FL_RAGE and V_C1_C2 in blue (protection increased in FL_RAGE) and red (protection decreased in FL_RAGE) are depicted on the X-ray structure of V_C1_C2 4YBH^16^. The differential plot of deuterium uptake degrees of peptides is in Supplementary materials Fig. S6.

The characterization of FL_RAGE indicated three regions in the V and C1 domain, which became stabilized (Fig. 7). Of note, these regions form the structural core of the protein and showed already strong protection in V_C1_C2, and in the case of FL_RAGE, became even more protected. The first region (R^77^**VLP**NGSL**FLP**AVG^90^) in the V domain contains six hydrophobic key residues (marked bold), which has been indicated as the oligomerization interface of the V-V domain (see Fig. 3a in^34^). Similarly, Yatime and Andersen^22^ designated the amino acids Leu79, Pro80, and Pro87 as important for the formation of a hydrophobic pocket that takes part in the stabilization of V_C1_C2 oligomers. Thus, V-domain oligomerization seems to be enhanced in higher order oligomers. The other two regions, which showed increased protection in FL_RAGE over V_C1_C2, were identified in the C1 domain, namely L^133^TAGVPNKVGTC^144^ and F^186^TLQSEL^192^. Although these two peptides are distant in protein sequence, they are in close proximity to each other in the crystal structure of V_C1_C2 (Fig. 7), forming the β-strands S11, S12, and S17a on the surface of a β-sheet, distal to the V domain and near to the flexible C1-C2 linker. Of interest, S11 has previously been found to undergo stabilization during dimer formation, obtained by C-terminal tyrosine crosslinking in exRAGE^23^. This evidence indicates that the C1 region participates in the formation of both dimers and higher-order oligomers. This three strands also face each other in the C1-C1 interface of the structural model of the heparin sulphate–stabilized hexamer (see Fig. 4a,b in^34^).

Moreover, we observed regions deprotected in FL_RAGE relative to V_C1_C2. In the C2 domain, we observed an increased rate of deuterium uptake for the proline-rich region W^271^MKDGVPLPLPPSPVL^286^, in which the residues LPL form a γ-turn. This finding indicates a significant destabilization in a region of the C2 domain, possibly resulting from an allosteric effect caused by the oligomerization of FL_RAGE. Moreover, this proline-rich region has been shown to form a loop on the external surface of the hexameric model mentioned above (Fig. 5d in^34^).

Changes in deuterium uptake were spread along the entire sequence, yet predominantly observed in the V-C1 region. Intermolecular-mediated contacts in the TM domain seemed to shift the oligomeric equilibria towards higher-order oligomers, resulting in a stabilization of regions that have previously been indicated as oligomerization contact sites. Their synergistic action seems to stabilize higher-order oligomers, as observed, for instance, in the heparan-stabilized hexamer structure^34^. The TM domain itself was also highly protected in FL_RAGE, unsurprisingly, as its amide hydrogens form a system of interacting intramembrane helices which are surrounded by detergent.

### Collision-induced unfolding (CIU) of V_C1, V_C1_C2, and FL_RAGE

**Figure 8:**
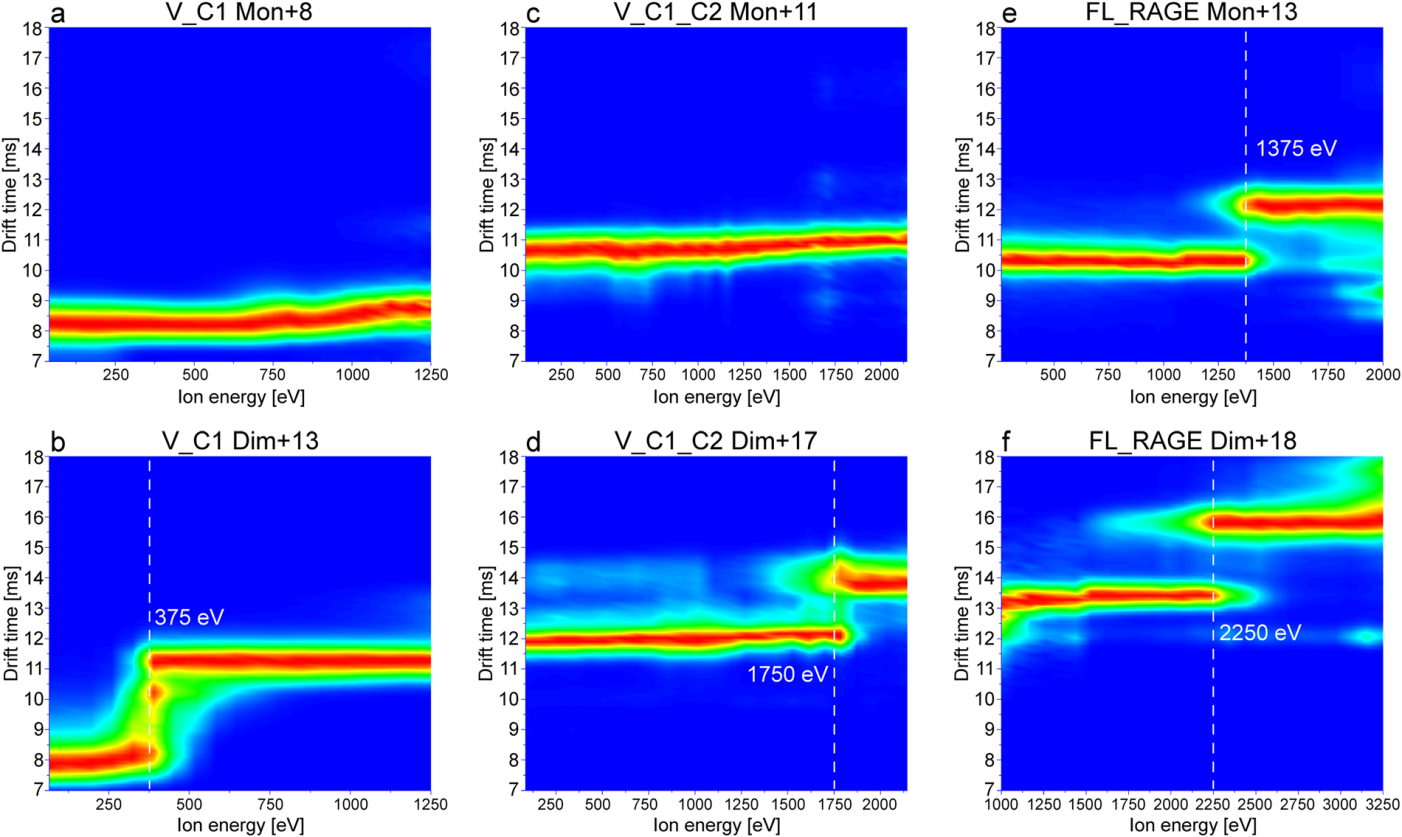
CIU plots of monomeric (**a**,**c**,**e**) and dimeric (**b**,**d**,**f**) species of the constructs V_C1, V_C1_C2, and FL_RAGE. The monomeric species of V_C1 (+8; m/z range 2290–2291) and V_C1_C2 (+11; m/z range 3174.3–3175.3) did not show unfolding in this energy range, while the dimeric species showed unfolding at 375 eV (+13; m/z range 3522–3523) and 1750 eV (+17; m/z range 4113–4115), respectively. FL_RAGE exhibited unfolding at 1375 eV for the monomer (+13; m/z range 3215–3217) and 2250 eV for the dimer (+18; m/z range 4642–4644). The relatively higher ion energy necessary for the unfolding of V_C1_C2 and FL_RAGE as compared to V_C1 indicates a stabilizing effect of the C2 and TM domain in the protein.

Recording ion mobility (IM)-MS along with a stepwise increase of collision energy, known as collision-induced unfolding (CIU)^35^, provides a useful tool for the characterization of the fold stability and domain architecture of proteins, as well as the stability of their dimerization interface. For CIU, protein ions are subjected to increasing activation energies in the collision cell prior to IM-MS separation, which enables the observation of gas-phase unfolding events. We performed such experiments for the constructs V_C1, V_C1_C2, and FL_RAGE in order to investigate the effect of different domains on the conformational stability and dimerization interface of the proteins. Figure 8 summarizes the obtained CIU fingerprints for monomeric and dimeric species of V_C1, V_C1_C2, and FL_RAGE, where the ion energy, defined as the ion’s charge state multiplied by the collision energy, is plotted against the drift time of the ions. Of interest, the monomers of V_C1 (Fig. 8a) and V_C1_C2 (Fig. 8c) did not show any noticeable unfolding, suggesting a quite stable conformation. The dimers of V_C1 (Fig. 8b) and V_C1_C2 (Fig. 8d), however, exhibited unfolding but with a much higher activation energy required for V_C1_C2 (1,750 eV) compared to V_C1 (375 eV).

Investigating micelle-embedded membrane protein complexes (e.g. FL_RAGE) with native MS requires part of the activation energy for the stripping of detergent molecules in order to release the membrane protein from this hydrophobic micellar environment^36^. Thus, only the remaining part of the collision energy is available for CIU. In the case of FL_RAGE, the release of the dimer required ca. 30 V, i.e. 540 eV based on the +18 dimer, of collision energy in addition to 200 V of activation using the sampling cone. Taking these conditions for the liberation of FL_RAGE into account, we can conclude that the thresholds for unfolding of both V_C1_C2 dimer (1,750 eV; Fig. 8d) and FL_RAGE dimer (2,250 eV; Fig. 8f) are relatively similar and much higher compared to the dimeric V_C1 (375 eV; Fig. 8b), indicating a considerable effect of the C-terminal C2 and TM domain on the stability of the dimer. The comparison of CIU plots of monomeric V_C1, V_C1_C2 and FL_RAGE highlights unfolding exclusively in the case of FL_RAGE at 1,375 eV (Fig. 8e), which suggests structural changes in the TM and CT regions that may weaken the stability of the fold.

## Discussion

Oligomerization has been implicated in numerous studies as a RAGE receptor signal transduction mechanism^19,20,22,34^. *In vitro*, structural aspects of oligomerization have been studied mainly for different variants of the extracellular parts of RAGE^13,16,20,22,23,34,37^, whereas the impact of TM and cytoplasmic tail have not been assessed. However, in many cases, oligomerization tendencies may be encoded in the TM parts. Mimicking the native properties of the target protein complex requires including this region. Here, we report a basic analysis of full-length RAGE oligomerization in comparison with its different truncated variants. We show major differences in oligomerization properties between full-length RAGE and separated extracellular RAGE domains. The analysis of truncated RAGE variants indicated that the C-terminal parts of the protein are more prone of forming oligomers as compared to the N-terminal domains. The results indicate that both the C2 domain and the TM region are crucial for the formation of integrated, intrinsically oligomeric native structural units of the receptor, building the flexible scaffold necessary for interaction with a variety ligands, and triggering ligand-dependent signal transduction.

Oligomerization of membrane receptors may be induced by ligands but can be constitutive. Apparently, RAGE belongs to the second group^38,39^, although the formation of the protein–ligand complex may further affect the structure and stoichiometry of the receptor complex. Structural studies of different truncated extracellular RAGE variants in the absence of ligands have shown different oligomerization modes in which different domains in different arrangements mediate self-association. Yatime and Andersen^22^ proposed V-V domain dimerization based on analysis of the X-ray structure of the extracellular part of RAGE (V_C1_C2). A similar type of oligomerization through the V domain has also been observed in solution^40^. A different mode of dimerization, with side-by-side contacts between V_C1 molecules and mainly involving residues in the C1 domain, was found in the X-ray structure of V_C1 protein^13^. In contrast to these V-V and C1-C1 modes of oligomerization, an NMR-based study of the soluble form of exRAGE (V_C1_C2) demonstrated an asymmetric type of dimer formation, mediated by both V_C1 and C2 interaction^20^. The evaluation of the oligomeric status of V_C1_C2 expressed in mammalian cells was carried out by native PAGE, which showed a prevalence of oligomers for V_C1_C2^19^. This observation contrasts with results obtained for V_C1_C2 *in vitro* that predominantly formed a monomer in solution^23,28,34,40^.

In the presence of ligands, diverse arrangements of the domains have also been found. Results of Small Angle X-ray Scattering in combination with crystallographic studies revealed, for instance, that in the presence of heparan sulphate, V_C1_C2 exists as a hexamer^34^. In this model of the configuration of the receptor complex, positively charged V domains interacting with negatively charged heparan sulphate form the central part of a wheel-like structure, in which the C1 and C2 domains form the spokes and rim of the wheel, respectively. Therefore, all domains can participate in the interaction network, which is in agreement with current results. Studies of complexes between RAGE and proteins of the S100 family have shown different models of RAGE oligomerization, mediated by ligand binding. X-ray structure analysis of the interaction between the V_C1_C2 and S100A6^16^ revealed that the homodimer of S100A6 binds two molecules of V_C1_C2, mainly through the C1 domain and a linker between C1 and C2 domains. Of interest, a completely different model of the RAGE S100 complex was proposed for the RAGE:S100B interaction^20^ in which the S100B homodimer bridged two preformed, asymmetrical RAGE homodimers. Taken together, these results indicate a multimodal type of RAGE oligomerization, required for recognition of different ligands^22^. Ligand-induced transitions between different oligomeric modes may be important in the regulation of ligand-specific signal transduction.

The role of the TM domain upon oligomerization of RAGE has not previously been characterized using high-resolution techniques. Yatime and Andersen^22^ suggested an involvement of the TM region in RAGE oligomerization. Thus, the TM region likely supplements or modifies the oligomerization of the extracellular parts of the protein^41^. Mimicking the native state requires this region to be taken into account. Studies of the oligomerization of RAGE using cross-linking of overexpressed FL_RAGE in mammalian cells have shown that the protein may exist as monomers or higher-order oligomers, although the dimeric state appears to be the prevalent oligomeric form^19,42^. These results agree with the observation that RAGE can form a disulphide-mediated dimer in mammalian cells^19^. In such a case, the structural unit of the RAGE receptor would be the disulphide-mediated dimer, where cysteines of the C2 domain form intermolecular bridges. Indeed, our data confirm that such a scenario is possible for C2 and C2_TM_CT (observed by non-reducing SDS-PAGE), in which we have observed a fraction of disulphide-linked dimers (Figs. 5g, 2f and S2b). In contrast, V_C1, V_C1_C2 (Fig. 2b,d), and FL_RAGE (Fig. 4b) did not reveal covalent dimers after non-reducing denaturation. C2 and C2_TM_CT were produced by cytoplasmic expression in *E. coli* where numerous reductases and reducing agents, such as reduced glutathione, prevent oxidation of cysteines and therefore the formation of disulphide bridges occurs later during protein purification in neutral/alkaline buffers. The C2 domain has the propensity to form a stable non-covalent dimer, in which intermolecular disulphides may be thermodynamically favored over intramolecular ones. V_C1 was also obtained by cytoplasmic expression in *E. coli*, however, in contrast to C2 and C2_TM_CT, this variant did not form a disulphide-mediated dimer. This lack of dimerization may be explained by its weaker oligomerization tendencies promoting intra- rather than inter-domain disulphides. Cytoplasmic expression of V_C1_C2 and FL_RAGE resulted in disulphide-mediated aggregates (Supplementary Fig. S2). Using periplasmic *E. coli* expression, described earlier for V_C1_C2^28^, allowed us to overcome this issue, leading to the formation of only intramolecular disulphide bonds^43^ (Supplementary Table S1).

It has become possible, for the first time, to observe the oligomerization of the full-length protein. We obtained a native MS spectrum of FL_RAGE, acquired at a relatively high AmAc concentration (600 mM), that displays abundant oligomeric signals up to tetramers. In general, high ionic strength buffers are expected to weaken electrostatically driven oligomer formation^44,45^. Thus, the fact that oligomers of FL_RAGE remain despite the relatively high AmAc concentration suggests a significant hydrophobically driven oligomerization in the TM domain of the protein. Besides that, interactions between extracellular domains, also of hydrophobic nature, may help to further stabilize the oligomeric complexes, which is indicated by native MS (Figs. 4a and 6), HDX (Fig. 7) and CIU (Fig. 8). In addition to hydrophobic effects, synergistic electrostatic interactions influence the oligomerization of RAGE.

## Conclusion

Our data showed differences in oligomerization tendencies for different variants of RAGE and revealed that oligomerization interfaces are spread along the RAGE sequence. The strongest oligomerization was observed in the C-terminal part of the protein, with significant involvement of the TM region. This finding suggested that both the C2 and TM domain are major factors for the stabilization of RAGE oligomers. At the same time, the increased protection in the V and C1 domain, in the context of full-length RAGE, as well as the dimer formation observed for V_C1 and V_C1_C2, indicate that the V and C1 domain also participate, at different levels, in oligomerization and that each domain plays a specific role.

## Materials and Methods

### Protein expression and purification

The production of recombinant proteins and mutagenesis are described in Supplementary information.

### Disulphide bond identification

Disulphide bond identification is described in Supplementary information.

### SEC-MALS analysis

The SEC-MALS setup consisted of an Agilent HPLC 1260 (including degasser, quaternary pump, autosampler, column holder, and UV-VIS diode array detector) in line with a DAWN-HELEOS multi-angle laser light-scattering detector and Optilab-T-rEX relative differential interferometer (Wyatt Technology). Samples were monitored at wavelengths of 280, 254, and 215 nm. One hundred microliters of protein samples were loaded onto a Superdex 200 Increase 10/300 column (GE Healthcare) equilibrated with buffer (25 mM Tris–Cl, 0.2 M NaCl, pH 8.0). Samples were run at room temperature at a flow rate of 0.5 ml/min. Agilent software was used to control the HPLC, and Wyatt Astra software was used for data collection and analysis. The results were analysed with the ASTRA software (Wyatt Technology) in accordance with the manufacturer’s instructions. For FL_RAGE and C2_TM_CT proteins, a buffer containing two times the critical micellar concentration (CMC) DDM was used. Because the TM domain of FL_RAGE and C2_TM_CT was surrounded by a DDM micelle, for molecular weight calculation, we used the “protein conjugate” module of the ASTRA software, which allows calculation of protein and detergent micelle molecular weights^25^. This method requires values of dn/dc for proteins and detergent and A280 for detergent and proteins. The value of dn/dc_protein_ is approximately the same (around 0.187 ml/g) for most soluble proteins. The values of dn/dc_DDM_ = 0.1435 ml/g and A280_DDM_ = 0.04 were taken from the (https://www.anatrace.com/Products/Detergents/MALTOSIDES/D310A). A280 for proteins was calculated using the ProtParam Tool (http://us.expasy.org).

### HDX-MS

Analysis of the H/D exchange was performed as described previously^23^ with modifications of the data analysis procedures. Peptides were identified using ProteinLynx Global SERVER software (PLGS, Waters). The list of identified peptides generated from the on-line pepsin digestion is presented in the supplementary data (Supplementary Table S3). Identified peptides after exchange were analysed by the DynamX 3.0 program (Waters) with the following acceptance criteria: minimum intensity threshold of 3000, minimum products per amino acid of 0.2, RT deviation ± 10S, and m/z deviation ± 10 ppm. The isotopic envelopes of the peptides after exchange were analysed using DynamX 3.0 with manual corrections wherever necessary. Final data containing molecular weights of all peptides at a given incubation time (*M*_*ex*_) were exported to Excel (Microsoft Office) for calculations. Percent of peptide deuteration was calculated with a formula that takes into consideration the molecular weights from the minimum (${M}_{ex}^{0}$) and maximum exchange (${M}_{ex}^{100}$) values of a given peptide. These values were obtained in control experiments as described previously^23^.

$$D( \% )=\frac{{M}_{ex}-{M}_{ex}^{0}}{{M}_{ex}^{100}-{M}_{ex}^{0}}\ast 100 \% $$

Error bars for percent deuteration D(%) were calculated as the square root of the sum of the standard deviation values of the compared states of three independent experiments.

To calculate the overall degree of protection of a given peptide (aggregated protection, show in Fig. S6a), we used kinetic plots showing the fraction of exchange at different incubation times. These plots allow monitoring of the increase in deuteration fraction over time in each peptide. For fully flexible regions, the exchange is instantaneous in the timeframe of the experiment, and the kinetic curve, linking the datapoints at different incubation times (1 minute, 30 minutes, 24 hours), is parallel to the horizontal axis at 100% of exchange. For fully protected regions, the kinetic curve is parallel to the horizontal axis at 0% exchange. To characterize the overall protection by a single number, we calculated the area over the kinetic curve, obtaining a degree of protection in % (aggregated protection), usually a number between 0% of protection in fully flexible regions and 100% of protection of fully protected (non-exchanging in the time frame of the experiment) regions. These numbers were further used to calculate the differential aggregated protection (ΔHDex) by subtraction of the aggregated protection in common peptides of FL_RAGE and V_C1_C2. Estimation of the error in the difference in exchange (ΔHDex), obtained by subtracting the aggregated protection in the FL_RAGE and V_C1_C2, was calculated as the square root of the sum of the standard deviation values of the compared states. Final graphs were plotted using OriginLab 9.

### Native MS

The samples were desalted and buffer-exchanged using P-6 Micro BioSpin columns (Bio-Rad) pre-equilibrated with 100 mM AmAc for V_C1 and C2, 500 mM (AmAc) for V_C1_C2, or 200 mM (AmAc) and 0.02% (w/v, 2 CMC) DDM for the C2_TM_CT wild-type and mutant proteins. The AmAc concentration for FL-RAGE was ranged from 200 to 1,000 mM containing either 0.04% (w/v, 2 CMC) Triton X-100 or 0.02% (w/v, 2 CMC) DDM. The concentration of all proteins was approximately 10 μM. MS measurements of all protein variants were also conducted under denaturing conditions achieved by the addition of 50% acetonitrile and 0.1% formic acid. Samples were introduced into the mass spectrometer by nano-electrospray ionization using in-house produced gold-coated glass capillaries at a voltage of +1.6 kV. MS experiments were performed either on a travelling-wave ion mobility Q-TOF (Synapt G2 HDMS, Waters, Manchester, UK)^46,47^ or a high-mass modified Q-TOF (MS Vision, Almere, NL)^48^ mass spectrometer tuned to maintain the native structure of soluble proteins and preserve the micelle-embedded membrane protein complexes upon entry into the gas phase. MS settings were adjusted to obtain an optimal ion transmission as follows: 1.4–1.6 kV capillary voltage, 25–75 V sampling cone, 3 V extractor cone, 30 °C source temperature, 15–30 V trap collision energy, and 5 V transfer collision energy. Gentle release of C2_TM_CT and FL_RAGE membrane protein complexes was achieved by the following instrument settings: 1.6–1.8 kV capillary voltage, 200 V sampling cone, 5 V extractor cone, 30 °C source temperature, 50–200 V trap collision energy, and 5 V transfer collision energy. Pressures throughout the instrument were 6–9 mbar backing, 3.1 mbar in the ion mobility cell, and 2.5 10^−2^ mbar for the trap and transfer cell, respectively. IM-MS was performed with a constant wave height of 40 V and a wave velocity of 800 m/s. CIU experiments were performed by stepwise (5 V) ramping the collision energy between 5–175 V for V_C1 and V_C1_C2 or between 85–175 V for FL_RAGE. Deconvolution of native mass spectra was performed using the online tool (http://www.bioprocess.org/esiprot/esiprot_form.php) and UniDec software^49^.

### AUC

Sedimentation velocity AUC experiments were performed using a ProteomeLab XL-I analytical ultracentrifuge (Beckman-Coulter, Indianapolis, USA), equipped with an An-50, 8-hole analytical rotor and double-sector charcoal-Epon cells (12-mm path length). The experiments were carried out at 20 °C, at 40,000 rpm (extracellular truncated variants V_C1_C2, V_C1, and C2) and 42,000 rpm (FL_RAGE, C2_TM_CT, and mutants the containing TM and cytosolic domains), using continuous scan mode and radial spacing of 0.003 cm. Scans were collected with no intervals between scans, both in absorbance (280 nm) and interference mode. Cells were loaded with 400 μl of sample and 410 μL of buffer. In case of TM-containing proteins, standard sample buffer (25 mM Tris, pH 7.5, 200 mM NaCl; but not reference buffer) was supplemented by the addition of 0.03% of DDM. Solvent densities and viscosities were measured at 20 °C using an Anton Paar (Graz, Austria) DMA 5000 densitometer and Lovis 2000 M rolling-ball viscometer. Partial specific volume and extinction coefficients for proteins were calculated using SEDNTERP software (http://bitcwiki.sr.unh.edu/index.php/Main_Page). Extracellular truncated variants: buffer density = 1.00716 g/cm^3^, and viscosity = 1.017 mPas. Partial specific volume (V-bar) of V_C1_C2 = 0.73366 cm^3^/g; V_C1 = 0.73208 cm^3^/g; and C2 = 0.73204 cm^3^/g. Full-length and N-terminal truncated variants: buffer with DDM (0.02%), density = 1.00772 g/cm^3^, and viscosity = 1.012 mPas; reference buffer without DDM, FL_RAGE V-bar = 0.7324. Data were analysed using mostly the “Continuous c(s) distribution” model of the SEDFIT (version 16.1) program^50^, but in case of the C2 fragment, “Continuous c(s) with bimodal f/f_0_” procedure was useful. In all cases, confidence level (F-ratio) was specified to 0.68. The proteins before loading were diluted to absorbance 0.9 at 280 nm. After loading into the centrifuge and reaching a temperature of 20 °C, the cells were equilibrated for 1 hour. Centrifugation was monitored by both absorbance (280 nm) and interference optics.

## Supplementary information

Dataset_1.

Supplementary Information.

## Data Availability

The datasets generated during and/or analysed during the current study are available from the corresponding author on request.
