# Supplementary material for: Enhanced oligomerization of full-length RAGE by synergy of the interaction of its domains
Source: Sci Rep. 2019 Dec 30;9:20332. doi: 10.1038/s41598-019-56993-9 (PMC6937306; doi:10.1038/s41598-019-56993-9)
Supplement: Supplementary file 2 — Supplementary Information. [file 41598_2019_56993_MOESM2_ESM.pdf]

Supplementary information

Enhanced oligomerization of full-length RAGE by synergy of the interaction of its domains.

Alexander Moysa[1*], Dietmar Hammerschmid[2], Roman H. Szczepanowski[3], Frank Sobott[2,4], Michal Dadlez[1]

1. Institute of Biochemistry and Biophysics, PAN, Pawinskiego 5a, 02-109 Warsaw, Poland.
2. Biomolecular & Analytical Mass Spectrometry, University of Antwerp, Groenenborgerlaan 171, 2020 Antwerp, Belgium;
3. International Institute of Molecular and Cell Biology, Trojdena 4, 02-109 Warsaw, Poland.
4. Astbury Centre for Structural Molecular Biology and School of Molecular and Cellular Biology, University of Leeds, Woodhouse Lane, LS2 9JT Leeds, UK.

[*]*Corresponding author: Alexander Moysa, a.alexandrmoysa@gmail.com*

List of included materials:

Supplementary Methods;

Supplementary Figures: Figures S1, S2, S3, S4, S5, S6, S7;

Supplementary Tables: Tables S1, S2, S3;

Supplementary References.

Supplementary methods

Construction and mutagenesis of expression plasmids

Codon optimization and gene synthesis of FL_RAGE was performed by the GenScript. The V_C1, V_C1_C2, C2, C2_TM_CT, and FL_RAGE genes were cloned into the *E. coli* expression vector pET24 thus to provide the expressed proteins with C terminal histidine tag. The V_C1_C2 and FL_RAGE genes, covering amino acid positions 22 to 340 and 22 to 404, respectively, were amplified by polymerase chain reaction (PCR) using the primers of the sequences provided below in the Supplementary Materials. To maintain native-like redox conditions, instead of cytoplasmic *E. coli* expression of V_C1_C2 and FL_RAGE, we used a periplasmic expression system that allowed acquisition of correct pairing of disulphide bonds and avoidance of disulphide-mediated aggregate formation, as can be observed for cytosolic expression systems (Supplementary Fig. S2, lanes 8–10). Therefore, for both variants, the forward primer contained a sequence of the signal peptide of *OmpA E. coli*. For the other variants, V_C1 (23-223), C2 (235-325), and C2_TM_CT (227-404), a cytoplasmic type of expression was used. The N-terminal primers contained the *Nde*I restriction site, and the C-terminal oligonucleotides coded for the *Xho*I restriction site. PCR was performed using *Pfu* DNA polymerase (Thermo Fisher Scientific) following the manufacturer's protocol. The resulting PCR fragments were digested with *Nde*I and *Xho*I (Thermo Fisher Scientific). The fragments were ligated to a pET24 vector using T4 DNA ligase (Thermo Fisher Scientific) and the recombinant clones sequenced to confirm the inserts. Plasmids were transformed into the *E. coli* strain BL21(DE3) for expression. Frozen bacterial stocks were maintained at −80°C in LB medium containing 50 µg/ml kanamycin and 15% glycerol. In C2_TM_CT_M1, glycine residues in the GILGGLG peptide of the TM domain of the C2 protein were replaced by alanine. The C2_TM_CT_M2 variant was obtained by deletion of AILAAL region in the TM domain of the C2_TM_CT_M1 protein. Mutations in C2_TM_CT were generated as described[1], using designed primers.

Optimized sequence of FL_RAGE gene obtained from GenScript.

GCGCAAAACA TTACCGCGCG TATCGGCGAG CCGCTGGTGC TGAAGTGCAA AGGTGCGCCG AAGAAACCGC CGCAGCGTCT GGAGTGGAAG
CTGAACACCG GCCGTACCGA AGCGTGGAAA GTTCTGAGCC CGCAAGGTGG CGGTCCGTGG GACAGCGTGG CGCGTGTTCT GCCGAACGGT
AGCCTGTTCC TGCCGGCGGT TGGCATCCAG GATGAGGGTA TTTTTCGTTG CCAAGCGATG AACCGTAACG GCAAGGAAAC CAAAAGCAAC
TACCGTGTGC GTGTTTATCA GATCCCGGGC AAGCCGGAAA TTGTTGATAG CGCGAGCGAG CTGACCGCGG GCGTGCCGAA CAAAGTTGGT
ACCTGCGTGA GCGAAGGCAG CTATCCGGCG GGTACCCTGA GCTGGCACCT GGACGGCAAG CCGCTGGTGC CGAACGAAAA GGGTGTGAGC
GTTAAAGAGC AGACCCGTCG TCACCCGGAA ACCGGTCTGT TCACCCTGCA AAGCGAACTG ATGGTTACCC CGGCGCGTGG CGGTGACCCG
CGTCCGACCT TCAGCTGCAG CTTTAGCCCG GGTCTGCCGC GTCATCGTGC GCTGCGTACC GCGCCGATTC AACCGCGTGT TTGGGAGCCG
GTGCCGCTGG AGGAAGTGCA ACTGGTGGTT GAGCCGGAGG GCGGTGCGGT TGCGCCGGGC GGTACCGTGA CCCTGACCTG CGAAGTTCCG
GCGCAGCCGA GCCCGCAAAT TCACTGGATG AAGGATGGTG TGCCGCTGCC GCTGCCGCCG AGCCCGGTTC TGATCCTGCC GGAGATTGGC
CCGCAGGATC AAGGTACCTA TAGCTGCGTG GCGACCCACA GCAGCCATGG TCCGCAGGAG AGCCGTGCGG TTAGCATCAG CATCATTGAA
CCGGGTGAGG AAGGTCCGAC CGCGGGTAGC GTGGGCGGTA GCGGCCTGGG TACCCTGGCG CTGGCGCTGG GCATTCTGGG CGGTCTGGGT
ACCGCGGCGC TGCTGATCGG CGTTATTCTG TGGCAGCGTC GTCAACGTCG TGGTGAGGAA CGTAAAGCGC CGGAAAACCA AGAGGAAGAG
GAAGAGCGTG CGGAACTGAA CCAAAGCGAA GAACCGGAGG CGGGCGAAAG CAGCACCGGC GGCCCG

List of primers for truncated variants

Protein	Primer 1	Primer 2
FL_RAGE	5'-CTGATCATATGAAAAAAACCGCGATTGCGATTGCGGTGGCGTTAGCG GGCTTTGCGACCGTGGCGCAGGCGGGCGCGCAAAACATTACCGCG-3'	5'- CTGATCTCGAGCGGGCCGCCGGTGCT-3'
V_C1_C2	5'-CTGATCATATGAAAAAAACCGCGATTGCGATTGCGGTGGCGTTAGCG GGCTTTGCGACCGTGGCGCAGGCGGGCGCGCAAAACATTACCGCG-3'	5'-CTGATCTCGAGGGTACCCAGGCCGCTACCG-3'
V_C1	5'-CTGATCATATGGCGCAAAACATTACCGCGCGTATCGGCGAG-3'	5'-CTGATCTCGAGGGCGCGGTACGCAGC-3'
C2_TM_CT	5'-CTGATCATATGCTGGAGGAAGTGCAA-3'	5'-CTGATCTCGAGACCTTCCTCACCCG-3'
C2	5'-CTGATCATATGCCGCGTGTTTGGGAGCCGGTG-3'	5'- CTGATCTCGAGCGGGCCGCCGGTGCT-3'

Amino acid sequences of constructs

FL_RAGE

GAQNITARIGEPLVLKCKGAPKKPPQRLEWKLNTGRTEAWKVLSPQGGGPWDSVARVLPNGSLFLPAVGIQDEGIFRCQAMNRNGKETKSNYRVRVYQIPGKPEIVD
SASELTAGVPNKVGTCVSEGSYPAGTLSWHLDGKPLVPNEKGVSVKEQTRRHPETGLFTLQSELMVTPARGGDPRPTFSCSFSPGLPRHRALRTAPIQPRVWEPVPLEE
VQLVVEPEGGAVAPGGTVTLTCEVPAQPSPQIHWMKDGVPLPLPPSPVLILPEIGPQDQGTYSCVATHSSHGPQESRAVSISIIEPGEEGPTAGSVGGSGLGTLALALGI
LGGLGTAALLIGVILWQRRQRRGEERKAPENQEEEEERAELNQSEEPEAGESSTGGPLEHHHHHH

V_C1_C2

GAQNITARIGEPLVLKCKGAPKKPPQRLEWKLNTGRTEAWKVLSPQGGGPWDSVARVLPNGSLFLPAVGIQDEGIFRCQAMNRNGKETKSNYRVRVYQIPGKPEIVD
SASELTAGVPNKVGTCVSEGSYPAGTLSWHLDGKPLVPNEKGVSVKEQTRRHPETGLFTLQSELMVTPARGGDPRPTFSCSFSPGLPRHRALRTAPIQPRVWEPVPLEE
VQLVVEPEGGAVAPGGTVTLTCEVPAQPSPQIHWMKDGVPLPLPPSPVLILPEIGPQDQGTYSCVATHSSHGPQESRAVSISIIEPGEEGPTAGSVGGSGLGTLEHHHH
HH

V_C1

AQNITARIGEPLVLKCKGAPKKPPQRLEWKLNTGRTEAWKVLSPQGGGPWDSVARVLPNGSLFLPAVGIQDEGIFRCQAMNRNGKETKSNYRVRVYQIPGKPEIVDS
ASELTAGVPNKVGTCVSEGSYPAGTLSWHLDGKPLVPNEKGVSVKEQTRRHPETGLFTLQSELMVTPARGGDPRPTFSCSFSPGLPRHRALRTALEHHHHHH

C2

LEEVQLVVEPEGGAVAPGGTVTLTCEVPAQPSPQIHWMKDGVPLPLPPSPVLILPEIGPQDQGTYSCVATHSSHGPQESRAVSISIIEPGEEGLEHHHHHH

C2_TM_CT

PRVWEPVPLEEVQLVVEPEGGAVAPGGTVTLTCEVPAQPSPQIHWMKDGVPLPLPPSPVLILPEIGPQDQGTYSCVATHSSHGPQESRAVSISIIEPGEEGPTAGSVGG
SGLGTLALALGILGGLGTAALLIGVILWQRRQRRGEERKAPENQEEEEERAELNQSEEPEAGESSTGGPLEHHHHHH

Expression and purification of RAGE proteins in *E. coli*

For protein overexpression, a frozen glycerol stock of BL21(DE3) cells transformed with plasmid-encoding protein was streaked onto an LB agar plate containing 50 µg/ml kanamycin and grown overnight at 37°C. The resultant bacterial colonies were resuspended in 50 ml of LB Broth medium (Formedium) containing 50 µg/ml kanamycin and grown at 37°C with shaking at 160 rpm for 4 hours. A total of 16 ml of bacteria was used to inoculate 1 l of LB Broth medium containing 50 µg/ml kanamycin in 5-l Erlenmeyer flasks. The culture was grown at 37°C with shaking at 160 rpm until OD600 reached 0.7–0.8 and induced by adding Isopropyl β-D-1-thiogalactopyranoside to a final concentration of 1 mM. The culture then was incubated overnight at 160 rpm and 25°C. Bacteria were harvested by centrifugation at 5000 ×g in a centrifuge (Beckman Counter, Fullerton, CA, USA). The medium was removed and the cells resuspended in 100 ml buffer (0.05 M Tris–HCl, 0.3 M NaCl, and 1 mM PMSF, pH 8.0) per 4 l of culture. For solubilization of proteins containing a TM domain (FL_RAGE, C2_TM_CT), depending on the experiment, either 2% of Triton X-100 or DDM was used. The lysozyme treatment was carried out with 10 mg/ml of lysozyme for 30 minutes on ice. The sonication was done for 5 minutes while incubating on ice, using the manufacturer's protocol. Samples of TM-containing proteins were gently mixed overnight at 4°C before centrifugation.

The extracts were clarified by centrifugation for 30 minutes at 14,000 rpm at 4°C. Extracts after centrifugation were then mixed with 2 ml of Ni Sepharose 6 Fast Flow slurry (GE Healthcare), pre-

equilibrated with lysis buffer. Samples were incubated with Ni Sepharose slurry at 4°C on a shaker at low speed overnight. The Ni Sepharose was washed with 10 column volumes of lysis buffer and 10 column volumes of wash buffer (50 mM Tris–Cl, 1 M NaCl, 50 mM imidazole, pH 8.0). For FL_RAGE and C2_TM_CT proteins, the detergent was added to the buffer (Triton X-100 or DDM) at a concentration of 2 CMC. RAGE proteins were eluted with buffer (50 mM Tris–Cl, 0.5 M NaCl, 250 mM Imidazole, pH 8.0) and Triton X-100 or DDM at 2 CMC for FL_RAGE and C2_TM_CT. The protein-containing fractions were identified by SDS–PAGE, under reducing conditions, on 4%–15% polyacrylamide mini gels (Mini-PROTEAN® TGX™ Precast Gels, Bio-Rad). Prior to most applications, Ni–NTA fractions were concentrated and purified by SEC either Ion exchange chromatography. Purification FL_RAGE was carried out using HiTrap SP cation exchange column (GE Healthcare) at pH 7.4 (50 mM Tris–Cl, 0.2 M NaCl; 0.04% (w/v) of Triton or 0.02% (w/v) of DDM), a salt gradient from 150 mM to 1 M NaCl. For V_C1_C2 and V_C1 and C2, we used Superdex 75 10/300 GL (GE Healthcare) equilibrated with buffer (25 mM Tris–Cl, 0.2 M NaCl, pH 7.4). For FL_RAGE, C2_TM_CT, and mutants, we used Superdex 200 10/300 GL (GE Healthcare) equilibrated with buffer (25 mM Tris–Cl, 0.2 M NaCl, pH 7.4; 0.04% (w/v) of Triton or 0.02% (w/v) of DDM for FL_RAGE). Fractions containing pure protein were combined and concentrated using an Amicon Ultra Centrifugal Filter Unit (100K WMCO for FL_RAGE and C2_TM_CT; 10K WMCO for V_C1, V_C1_C2, and C2) to ~5 mg/ml and stored at 4°C. Protein concentration (except samples containing Triton X-100) was determined by absorbance at 280 nm with an extinction coefficient calculated from the amino acid composition using ProtParam Tool (http://us.expasy.org). The mutants C2_TM_CT M1 and M2 were expressed and purified as described above for the C2_TM_CT protein.

SDS_PAGE

The purified proteins were analysed by SDS-PAGE. Protein samples were mixed with 2x sample buffer with 200mM DTT and without for reducing and non-reducing conditions, respectively. After heating at 80°C for 10 minutes samples were loaded into gels. The absence of DTT enables S-S bonds to be retained, resulting in additional bands that correspond to disulphide-mediated oligomers. In the case of reducing conditions, the protein samples display only one band. Unstained Protein Molecular Weight Marker (Thermo Fisher Scientific) was used. Proteins were loaded on discontinuous gel system with 4–15% Mini-PROTEAN® TGX™ Precast Protein Gels Bio-Rad (a, b, e) and 5% (w/v) polyacrylamide loading gel and 12% (w/v) separating gel (c). Electrophoresis was conducted with a Bio-Rad mini protean tetra cell gel system under a continuous voltage of 120 V. Gels were stained with Coomassie brilliant blue staining solution.

Disulphide bond identification

As the distribution of trypsin cleavage sites in FL_RAGE does not allow the acquisition of all Cys–Cys dipeptides, we performed a pepsin digestion. In addition to an increasing number of obtained peptides[2], pepsin digestion has also the important advantage of preventing the formation of artificially reshuffled

disulphide bridges, which has been observed in the case of protein trypsin digestion under a pH range of 7.5–8.5[3]. Pepsin digests were collected from the Poroszyme™ Immobilized Pepsin Cartridge, 2.1 mm x 30 mm (Applied Biosystems™) dried and half of them dissolved in acidic buffer (6 M Gdn-HCl; 2 M glycine) for preventing disulphide bond reshuffling. Another half of the samples were dissolved in alkaline buffer (25 mM Tris-HCl, 6 M Gdn-HCl, pH 8.5) for further reducing cysteines by TCEP and iodoacetamide alkylation, following the recommendation of Gundry[4].

The peptide mixtures were analysed by LC-MS/MS using a Nano-Acquity LC system (Waters) and an Orbitrap Velos mass spectrometer. The peptide mixture was applied to an RP-18 trap (nanoACQUITY Symmetry® C18 – Waters 186003514) using 0.1% trifluoroacetic acid (v/v) in water as the mobile phase and then transferred to a nano-HPLC RP-18 column (nanoACQUITY BEH C18 - Waters 186003545) using an acetonitrile gradient (0%–60% acetonitrile in 100 min) in the presence of 0.05% formic acid with a flow rate of 250 nl/min. The column outlet was coupled directly to the ion source of the spectrometer working in the regime of data-dependent acquisition mode. For full-scan MS, we used a mass range of m/z 300–2000 and resolution of R = 60000. Then, up to 10 HCD MS/MS scans were acquired of the most intense at least doubly charged ions (resolution 30000, isolation window 4.0 m/z, normalized collision energy 40.0, dynamic exclusion 20.0 s). All spectra were recorded in profile mode.

Raw data from the mass spectrometer were converted to MGF (Mascot generic files) format with program msConvert GUI from ProteoWizard Toolkit version 3.0.9134[5]. We used the program pLink version 2.3.2[6] for cross-link identification. MS data in MGF format and FL_RAGE protein sequence in FASTA format were loaded into the program and the cross-linked residues searched. pLink search parameters were set as the following: precursor mass tolerance - 10 ppm, fragment mass tolerance - 10 ppm, cross-linker - disulfide bond SS, variable modification C - 57.02146, peptide length minimum 6 and maximum 60 amino acids per chain, peptide mass minimum 600 and maximum 6000 Da per chain, enzyme nonspecific.

Supplementary Figures

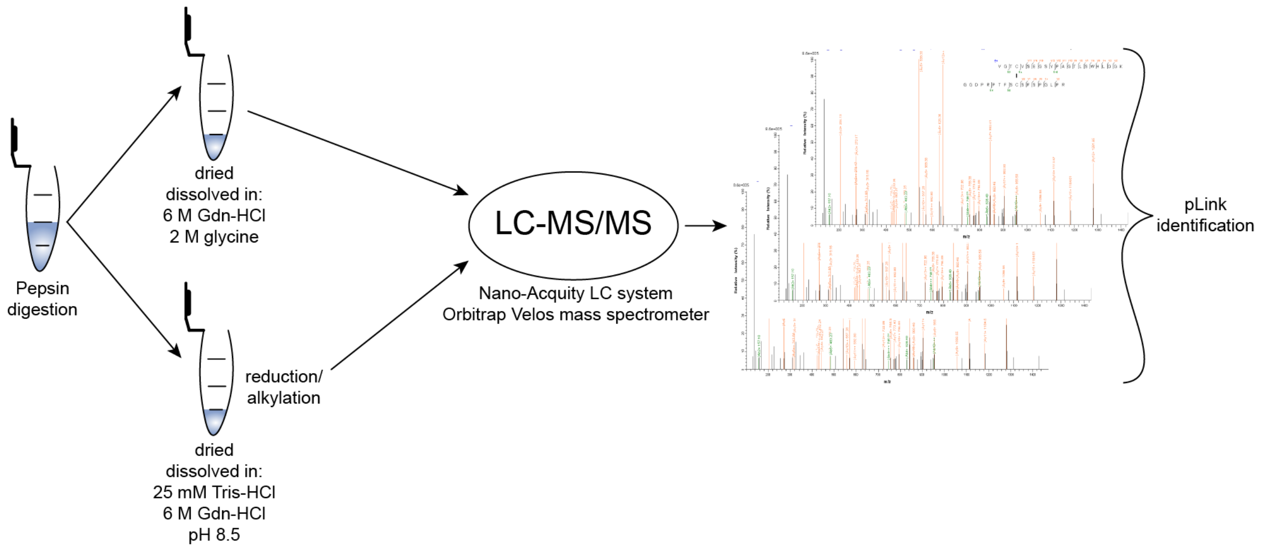

Fig. S1. MS-based strategy for disulphide bond analysis

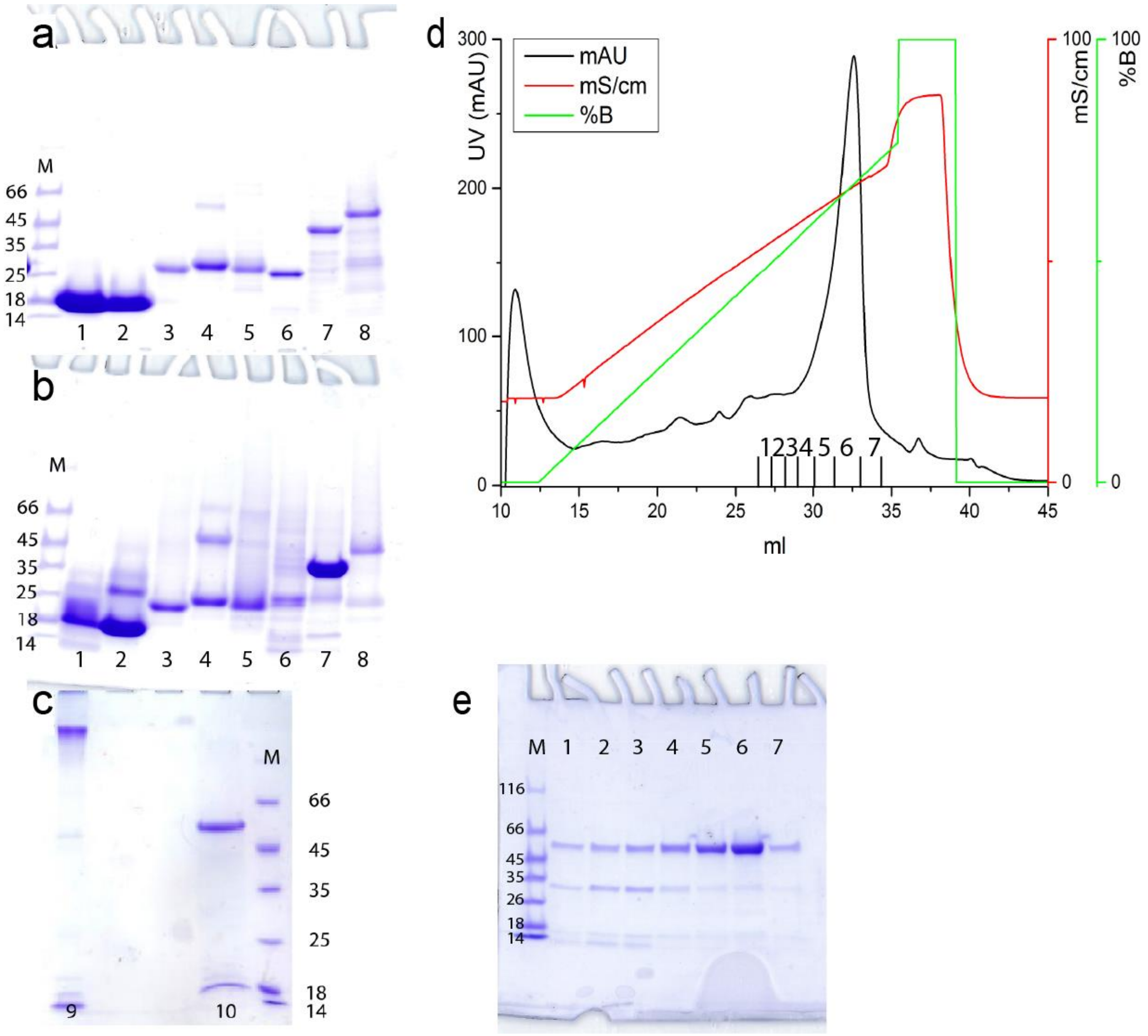

Fig. S2. SDS-PAGE of purified full-length RAGE and its truncated variants under reducing conditions (a) and nonreducing conditions (b). Lanes: 1 – monomeric fraction of C2 after SEC; 2 – dimeric fraction of C2 after SEC; 3 – C2_TM_CT Mutant 2; 4 – C2_TM_CT Mutant 1; 5 – C2_TM_CT; 6 – V_C1; 7 – V_C1_C2 periplasmic expression; 8 – FL_RAGE periplasmic expression. (c) FL_RAGE obtained after cytoplasmic expression (9 – without reducing agents; 10 – under reducing conditions). (d) Ion exchange chromatogram of FL_RAGE. (e) SDS-PAGE of collected fractions.

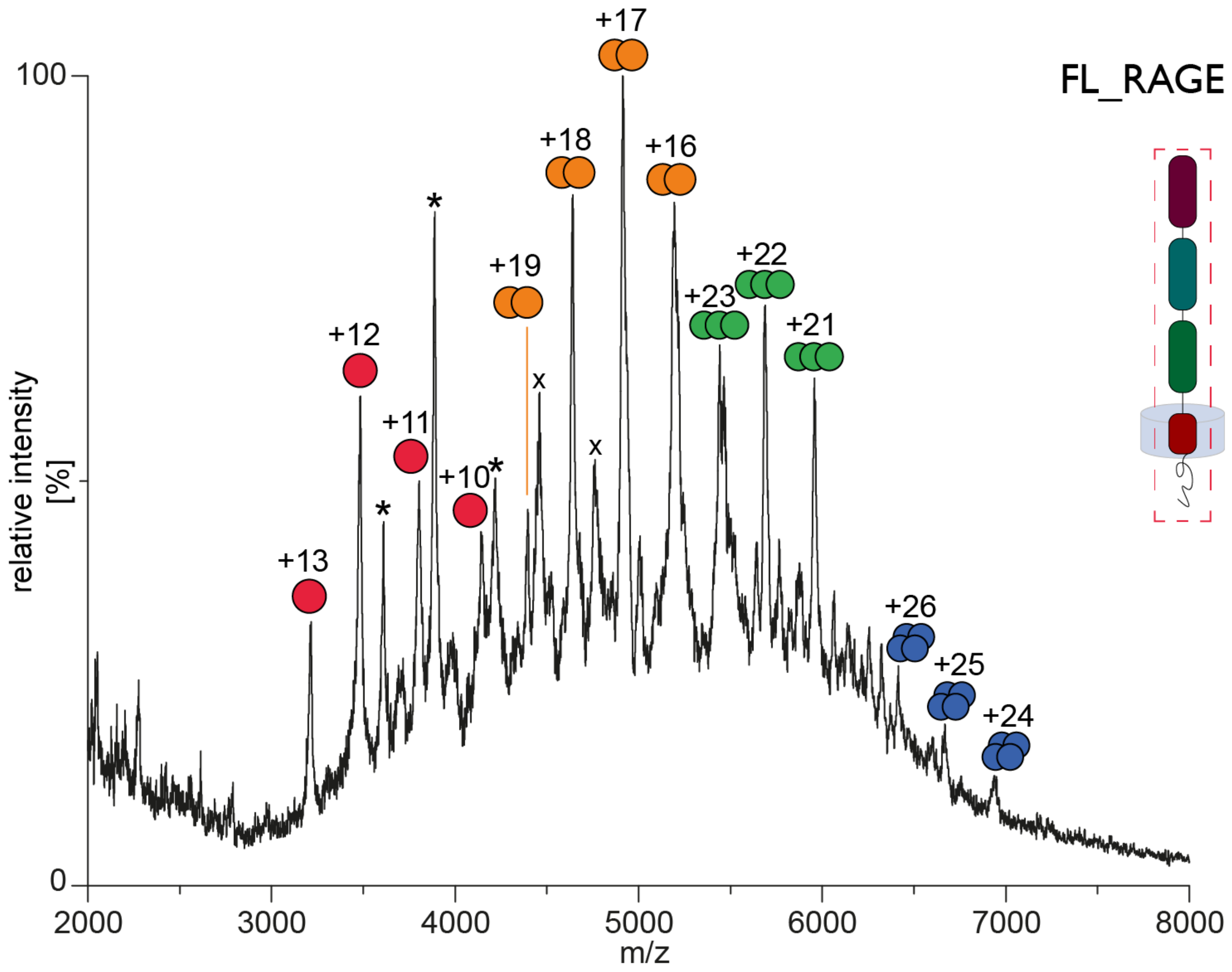

Fig. S3. Native MS of FL_RAGE. Other than in Figure 4, the purificaiton of FL_RAGE was performed with SEC instead of IEC. Peaks are marked by colored dots and the corresponding charge state for monomers and multimers of FL_RAGE accordingly. Two peak series in the area between 3,500 and 5,000 represent species of 50.5 kDa (*) and 71.4 kDa (x), which could not be assinged to FL_RAGE.

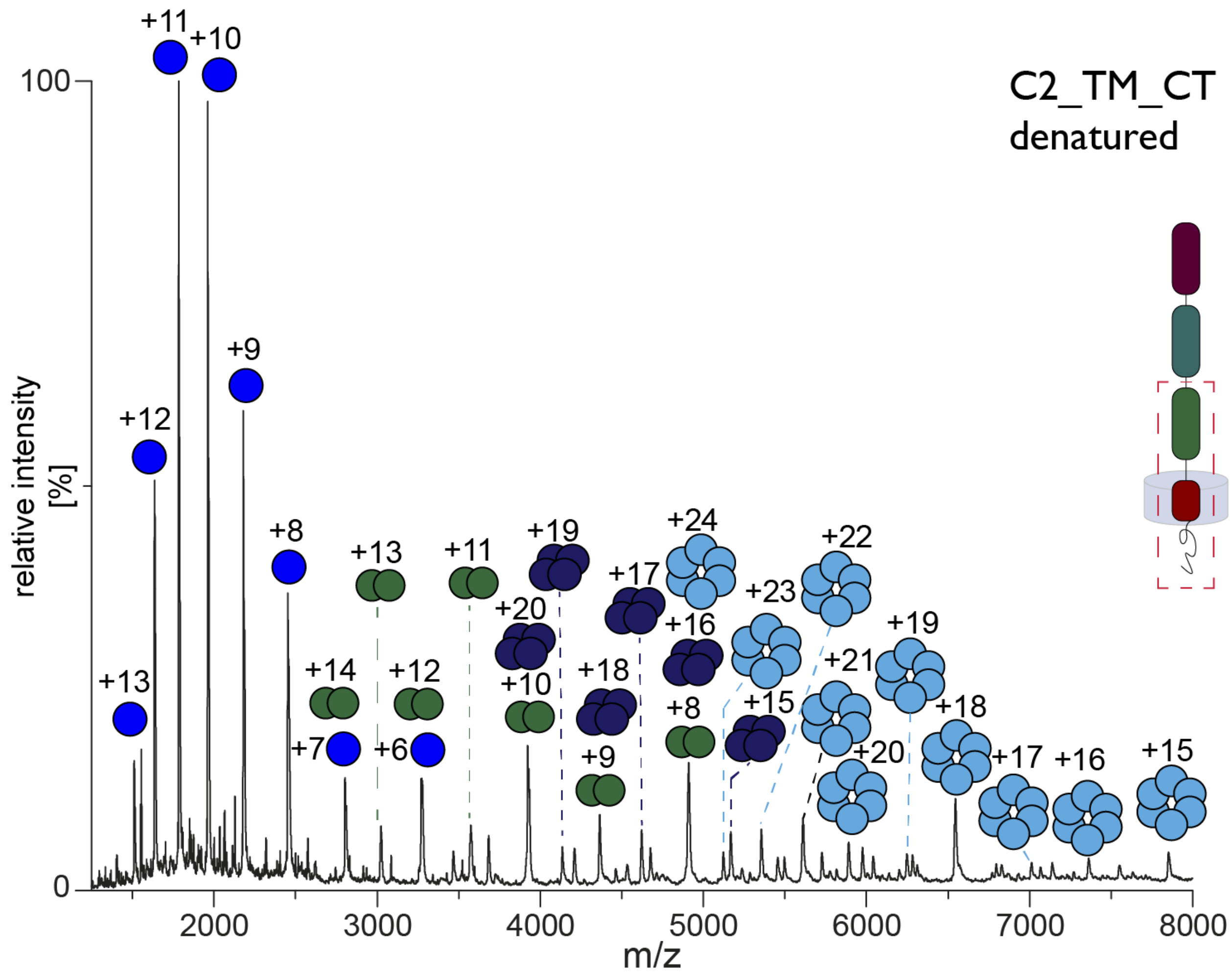

Fig. S4. Mass spectrum of C2_TM_CT under denaturing conditions. After the denaturation of C2_TM_CT (addition of 50% acetonitrile and 0.1% formic acid), we observed not only dimeric but also tetrameric and hexameric species.

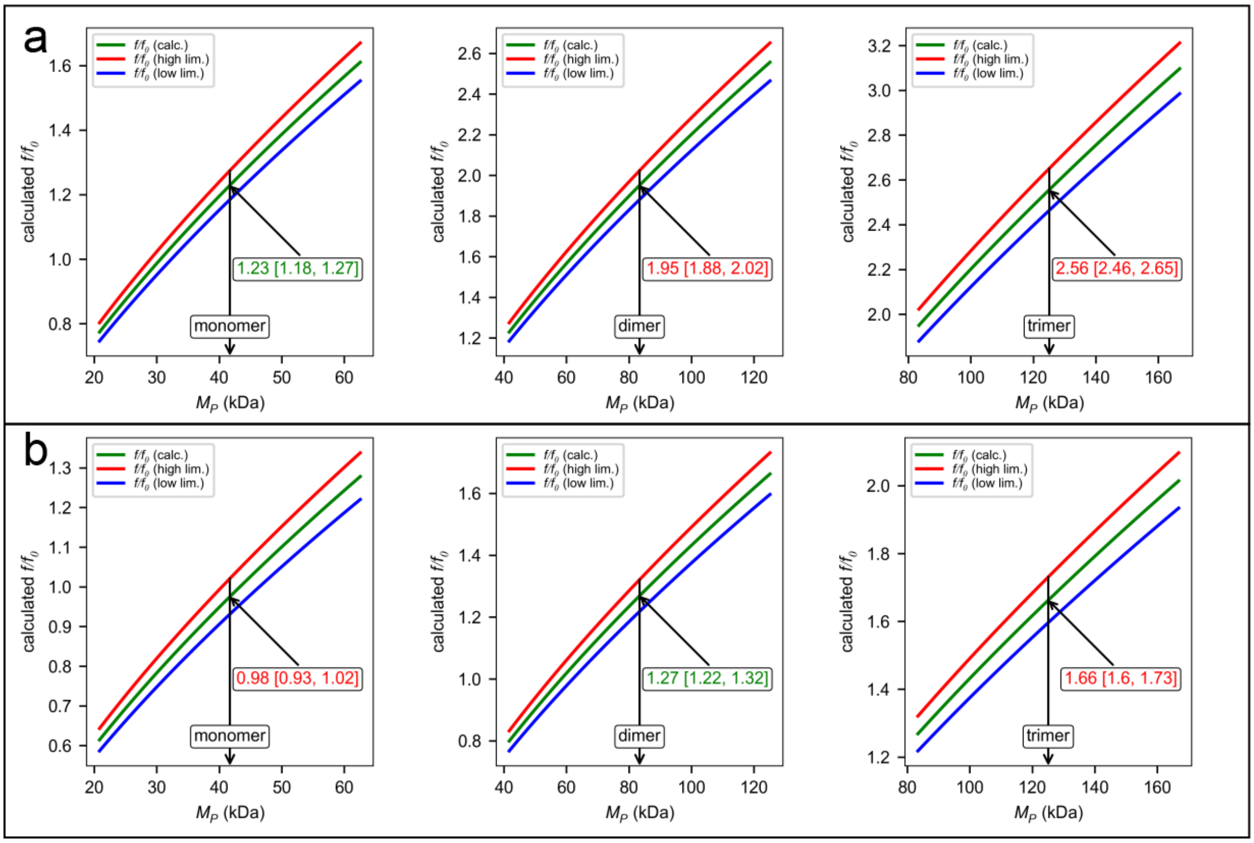

Fig. S5. Analysis of 4.4S and 7.4S peaks from the dataset of FL_RAGE (Fig. 3b) (5.8S peak was analyzed earlier) using f/fo calculation type of the GUSSI Membrane Protein Module. The analysis of the 4.4S peak indicates the presence of a monomer - only in this case, the f/fo values are within the anticipated range (a). For the 7.4S peak, the f/fo values for both the dimer (1.27) and the trimer (1.67) are within the allowable range (b).

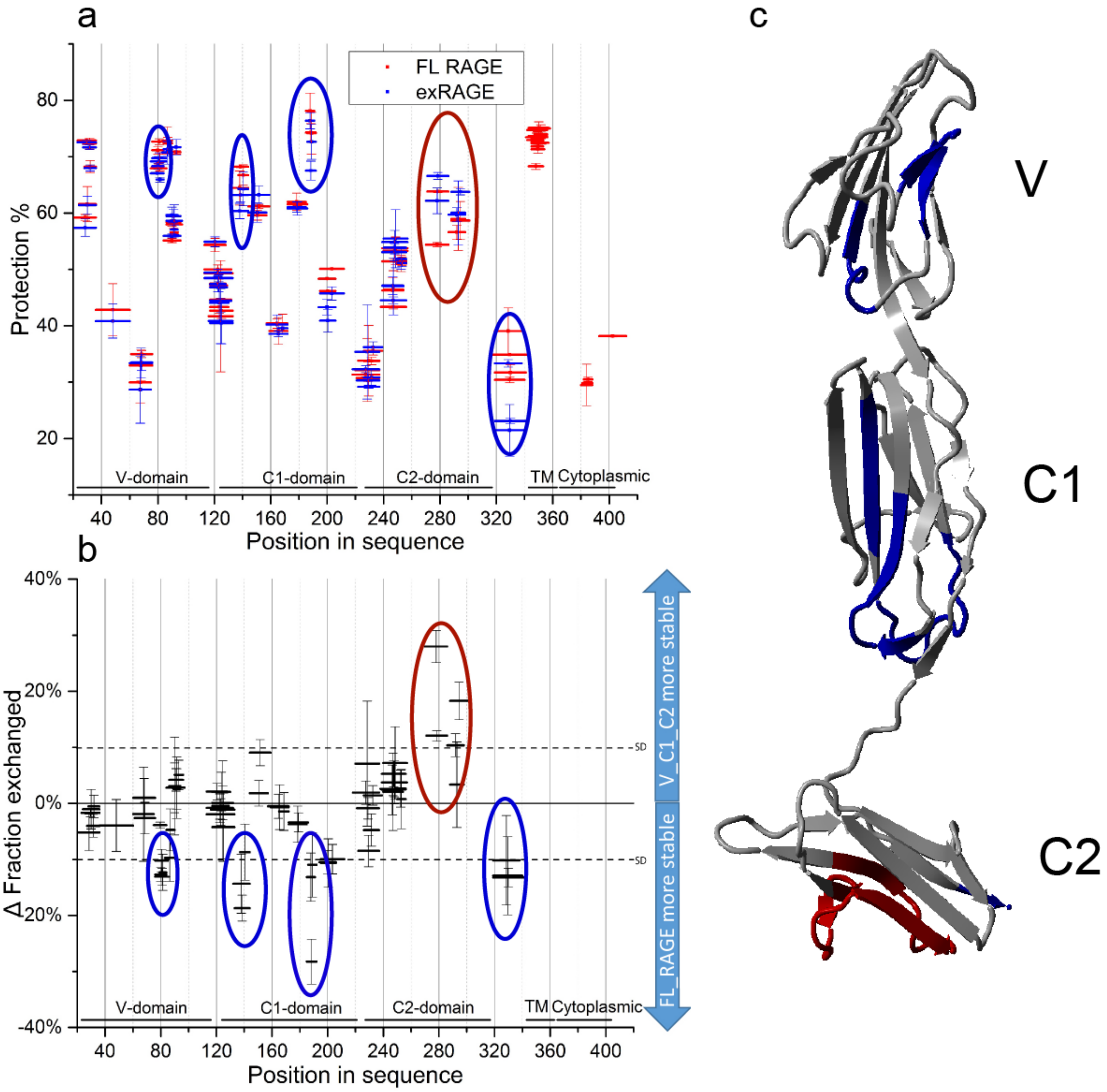

Fig. S6. Comparison of the HDX aggregated protection of FL_RAGE (red) and V_C1_C2 (blue) peptides (a). Regions characterised by a stable structure are indicated by a high level of protection while dynamic region show low level of protection. The position of a peptide is shown on the horizontal axis, represented by a horizontal bar with a length equal to the length of the peptide. Small differences in the exchanged fraction for overlapping peptides underscore the good internal consistency of the data. Y-axis error bars are standard deviations calculated from at least three independent experiments. For a large majority of peptides, the level of exchange did not differ significantly between FL_RAGE and V_C1_C2 and retained intertwining between relatively protected and completely unprotected regions. However, significant differences were detected at three protein regions of V and C1 domains, illustrated best in panel b, showing the results of subtracting the fraction of exchange in FL_RAGE and V_C1_C2 peptides (Δ Fraction exchanged). The subtraction procedure is described in Materials and Methods. Negative Δ values indicate increased protection in FL_RAGE. H/D exchange differences between FL_RAGE and V_C1_C2 in blue (protection increased in FL_RAGE) and red (protection decreased in FL_RAGE) are shown overlaid on the X-ray structure of V_C1_C2 4YBH (c).

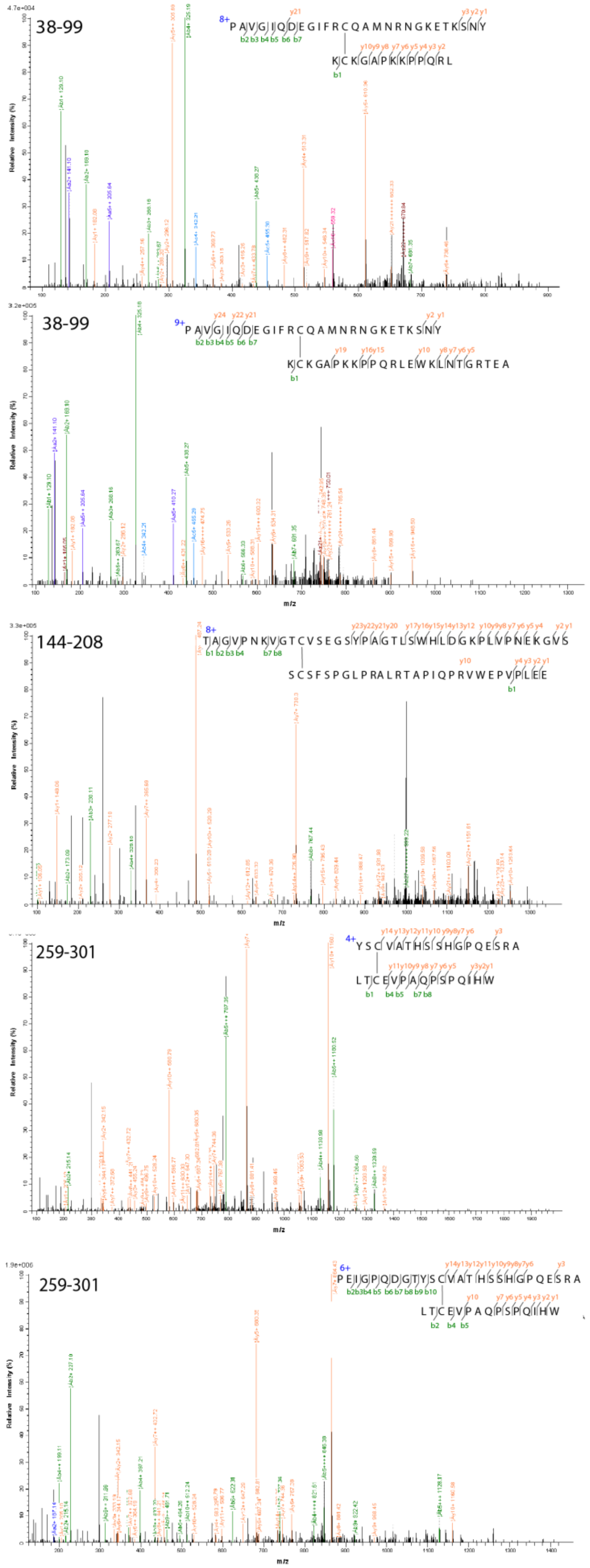

Fig. S7. Example of MS/MS spectra of identified crosslinked peptides.

Supplementary tables

Table S1. The number of observed dipeptides containing disulphide bridges after pepsin digestion. 182 hits were found by pLink (Dataset 1 and Fig. S7). A total number of 48 unique Cys–Cys dipeptides were observed.

Protease	Cys–Cys	Number of identified peptides
Pepsin	38–99	7
Pepsin	144–208	1
Pepsin	259–301	40

Table S2. The amino acid sequence of the TM domain of N-terminal truncated variants of RAGE. Glycine–alanine substitution and deletion of the region covering GxxxG motif were obtained by mutagenesis.

**Protein**	**Amino acid sequence of TM domain**
C2_TM_CT	GTLALALGILGGLGTAALLIGVILW
Mutant 1	GTLALALAILAALATAALLIGVILW
Mutant 2	GTLALAL ATAALLIGVILW

Table S3. The peptic peptide list of RAGE constructs. The list was obtained from LC-MS/MS analysis of the non-deuterated sample analysed by the PLGS program. Peptides are sorted according to their position in the protein sequence. Mass = molecular mass, RT = retention time.

Sequence	Start	End	Mass	RT
GAQNITARIGEPLVL	22	36	1551.885	6.17
QNITARIGEPLVL	24	36	1423.827	6.06
NITARIGEPLVL	25	36	1295.768	6.08
ARIGEPLVL	28	36	967.594	5.53
RIGEPLVL	29	36	896.556	5.52
KCKGAPKKPPQRLEWKLNTGRTEA	37	60	2736.509	3.01
WKVLSPQGGGPWDSV	61	75	1612.812	6.58
WKVLSPQGGGPWDSVA	61	76	1683.849	6.35
KVLSPQGGGPWDSVA	62	76	1497.770	5.26
ARVLPNGSL	76	84	926.542	4.09
ARVLPNGSLFL	76	86	1186.694	6.26
RVLPNGSL	77	84	855.505	3.94
RVLPNGSLF	77	85	1002.573	5.48
RVLPNGSLFL	77	86	1115.657	6.14
PNGSL	80	84	487.251	3.94
LFLPAVG	84	90	716.434	6.59
FLPAVG	85	90	603.350	4.94
FLPAVGIQDEGI	85	96	1258.668	6.53
PAVGIQDE	87	94	828.410	3.62
PAVGIQDEGI	87	96	998.515	5.02
PAVGIQDEGIF	87	97	1145.584	6.79
GIQDE	90	94	561.252	5.02
IQDEGI	91	96	674.336	4.02

IQDEGIF	91	97	821.404	6.21
RVRVYQIPGKPEIVD	114	128	1769.007	3.90
RVRVYQIPGKPEIVDSASE	114	132	2143.151	3.93
RVRVYQIPGKPEIVDSASEL	114	133	2256.235	4.69
VYQIPGKPEIVDSASEL	117	133	1844.964	5.42
YQIPGKPEIVD	118	128	1258.668	4.38
YQIPGKPEIVDS	118	129	1345.700	4.26
YQIPGKPEIVDSA	118	130	1416.737	4.44
YQIPGKPEIVDSASE	118	132	1632.812	4.35
YQIPGKPEIVDSASEL	118	133	1745.896	5.31
LTAGVPNKVGTC	133	144	1159.614	3.42
TAGVPNKVGTC	134	144	1046.530	2.85
PNKVGTC	138	144	718.355	2.85
VSEGSYPAGTLSW	145	157	1353.632	6.61
VSEGSYPAGTLSWHL	145	159	1603.775	6.70
HLDGKPLVPNEKGVS	158	172	1589.865	2.91
DGKPLVPNEKGVS	160	172	1339.722	2.91
PNEKGVS	166	172	730.373	2.91
VKEQTRRHPETGL	173	185	1550.840	2.10
VKEQTRRHPETGLF	173	186	1697.908	3.35
FTLQSE	186	191	724.351	4.54
FTLQSEL	186	192	837.435	6.22
TPARGGDPRPTF	195	206	1271.649	3.78
PARGGDPRPTF	196	206	1170.601	3.78
PARGGDPRPTFSCSFSP	196	212	1778.828	3.81
ALRTAPIQPRVWEPVPLEE	219	237	2201.208	5.58
RTAPIQPRVWEPVPLEE	221	237	2017.087	5.42
TAPIQPRVWEPVPLEE	222	237	1860.985	6.02
APIQPRVWEPVPLEE	223	237	1759.938	6.02
PRVWEPVPLEE	227	237	1350.705	5.65
PRVWEPVPLEEVQ	227	239	1577.832	5.98
QLVVEPEGGAVAPGGTVT	239	256	1680.880	4.55
LVVEPEGGAVAPGGT	240	254	1352.706	4.09
LVVEPEGGAVAPGGTVT	240	256	1552.822	4.30
LVVEPEGGAVAPGGTVTL	240	257	1665.906	6.12
VVEPEGGAVAPGGT	241	254	1239.622	3.24
VVEPEGGAVAPGGTVT	241	256	1439.738	3.55
VVEPEGGAVAPGGTVTL	241	257	1552.822	5.17
APGGTVT	250	256	602.314	3.55
PGGTVT	251	256	531.277	3.55
WMKDGVPLPLPPSPVL	271	286	1745.966	6.91
MKDGVPLPLPPSPVL	272	286	1559.887	6.27
ILPEIGPQDQGT	287	298	1267.653	4.86
PEIGPQDQGT	289	298	1041.485	3.22
PEIGPQDQGTYSC	289	301	1394.589	4.39
IIEPGEEGPTAGSVGGSGL	320	338	1726.849	5.04

IIEPGEEGPTAGSVGGSGLGT	320	340	1884.919	4.62
IIEPGEEGPTAGSVGGSGLGTL	320	341	1998.003	5.84
ALALGILGGLGTAAL	342	356	1310.804	8.50
LALGILGGLGT	343	353	984.609	7.72
LALGILGGLGTAAL	343	356	1239.767	8.38
ALGILGGLGT	344	353	871.525	6.98
ALGILGGLGTAAL	344	356	1126.683	7.82
ALGILGGLGTAALL	344	357	1239.767	8.64
GILGGLGT	346	353	687.404	5.77
GILGGLGTA	346	354	758.441	5.87
GILGGLGTAA	346	355	829.478	5.77
GILGGLGTAAL	346	356	942.562	6.99
GILGGLGTAALL	346	357	1055.646	7.88
GILGGLGTAALLI	346	358	1168.730	8.64
LGGLGT	348	353	517.298	7.72
LGGLGTAAL	348	356	772.456	8.37
EEEERAEL	381	388	1004.453	3.44
EEERAEL	382	388	875.411	3.28
EERAEL	383	388	746.368	3.15
PEAGESSTGGPLEHHHHHH	394	412	2052.902	2.15

Supplementary References

1. Liu, H. & Naismith, J. H. An efficient one-step site-directed deletion, insertion, single and multiple-site plasmid mutagenesis protocol. *BMC Biotechnol.* **8,** 91 (2008).

2. Liu, F., van Breukelen, B. & Heck, A. J. R. Facilitating protein disulfide mapping by a combination of pepsin digestion, electron transfer higher energy dissociation (EThcD), and a dedicated search algorithm SlinkS. *Mol. Cell. Proteomics* **13,** 2776–86 (2014).

3. Gorman, J. J., Wallis, T. P. & Pitt, J. J. Protein disulfide bond determination by mass spectrometry. *Mass Spectrom. Rev.* **21,** 183–216 (2002).

4. Gundry, R. L. *et al.* Preparation of proteins and peptides for mass spectrometry analysis in a bottom-up proteomics workflow. *Curr. Protoc. Mol. Biol.* **Chapter 10,** Unit10.25 (2009).

5. Chambers, M. C. *et al.* A cross-platform toolkit for mass spectrometry and proteomics. *Nat. Biotechnol.* **30,** 918–920 (2012).

6. Fan, S.-B. *et al.* Using pLink to Analyze Cross-Linked Peptides. in *Current Protocols in Bioinformatics* **49,** 8.21.1-8.21.19 (John Wiley & Sons, Inc., 2015).
